# Genetic, Clinical, and Management Characteristics of Duchenne Muscular Dystrophy in Saudi Arabia

**DOI:** 10.3390/healthcare14070857

**Published:** 2026-03-27

**Authors:** Abdulaziz S. AlSaman, Fouad Al Ghamdi, Ahmed K. Bamaga, Nahla AlShaikh, Mohammed Al Muqbil, Osama Muthaffar, Fahad A. Bashiri, Baleegh Ali, Hebah Qashqari, Elena Heider, Ahmad Itani, Abdullah A. Alshahrani, Mohammed A. Al Muhaizea

**Affiliations:** 1 Pediatric Neurology Department, National Neuroscience Institute, King Fahad Medical City, Riyadh 12231, Saudi Arabia; 2 Neuroscience Center, King Fahad Specialist Hospital, Dammam 32253, Saudi Arabia; 3 Neurology Division, Department of Pediatrics, Faculty of Medicine, King Abdulaziz University Hospital, King Abdulaziz University, Jeddah 21589, Saudi Arabia; 4 Department of Pediatrics, King Faisal Specialist Hospital and Research Centre, Jeddah 21499, Saudi Arabia; dr.h.qashqari@gmail.com; 5 Department of Pediatrics, Ministry of National Guard Health Affairs, Jeddah 22384, Saudi Arabia; 6 College of Medicine, King Saud bin Abdulaziz University for Health Sciences, Riyadh Campus, Jeddah 21423, Saudi Arabia; 7 King Abdullah International Medical Research Center, Jeddah 22384, Saudi Arabia; 8 College of Medicine, King Saud bin Abdulaziz University for Health Sciences, Jeddah Campus, Riyadh 22490, Saudi Arabia; 9 Division of Pediatric Neurology, King Abdullah Specialized Children’s Hospital, Ministry of National Guard Health Affairs, Riyadh 14611, Saudi Arabia; 10 King Abdullah International Medical Research Center, Ministry of National Guard Health Affairs, Riyadh 11481, Saudi Arabia; 11 Division of Pediatric Neurology, Department of Pediatrics, College of Medicine, King Saud University, Riyadh 11461, Saudi Arabia; 12 King Saud Medical City, Riyadh 11461, Saudi Arabia; 13 PTC Therapeutics, 6300 Zug, Switzerland; 14 King Faisal Specialist Hospital and Research Centre, Riyadh 11211, Saudi Arabia; abdulla.shahrani@gmail.com; 15 Neuroscience Centre, King Faisal Specialist Hospital and Research Centre, College of Medicine, Alfaisal University, Riyadh 11211, Saudi Arabia

**Keywords:** Duchenne muscular dystrophy, treatment management, progressive muscle degeneration, dystrophin gene

## Abstract

Background: Duchenne muscular dystrophy (DMD) is a rare, disabling, and life-threatening X-linked recessive disorder caused by mutations in the dystrophin gene. The current standard of care is treatment with corticosteroids, which aim to decrease inflammation-induced muscle damage and delay disease progression. Here, we aim to describe clinical, genetic, and diagnostic characteristics and evaluate current management practices of DMD patients in the Kingdom of Saudi Arabia (KSA). Methods: This was an ambispective (prospective and retrospective) observational multicenter study evaluating characteristics of patients aged 1–14 years with genetically confirmed DMD in the KSA. The variables of interest were demographics, genetic mutations, clinical characteristics, and initial management. The relationship between the age at diagnosis, initial management plan (standard of care), and age at initiation of treatment on disease outcomes was also evaluated. Results: A total of 226 patients (181 in the retrospective part and 45 in the prospective part) were enrolled. The most common type of genetic mutation was large deletions (134 patients, 59.3%). The median age of first symptom was 2.7 years (IQR: 2.0–4.6 years) and the median age at diagnosis was 7.0 years (IQR: 4.8–8.5 years). Among these patients, the most common initial symptoms were difficulty in walking (87.7%) and waddling gait (41%). The initial management plan for DMD patients involved medication (75.6%) and physical therapy (71.0%). The most frequently prescribed initial medications were vitamin D (82%) and corticosteroids (62.3%). In total, 6/226 patients (2.6%) received ataluren; they all had identified nonsense mutations. The median age of corticosteroid initiation was 7.1 years (IQR: 5.7–8.7). The median age at loss of ambulation (LoA) was 9.8 years (IQR: 8.0–11.4 years) in the non-treated patients; it was 10.1 years (IQR: 9.3–11.2 years) in the steroid-only group and 10.8 years (10.8, 10.8) in the combined ataluren and steroid treatment group. Discussion: Age of diagnosis and age of treatment initiation is relatively late in the KSA. However, early diagnosis and early treatment onset is associated with better clinical outcomes, mainly a delay in LoA. Therefore, there is an urgent need for raising awareness and enhancing early screening in the KSA.

## 1. Introduction

Duchenne muscular dystrophy (DMD) is a severe, progressive, and X-linked recessive disorder characterized by muscle weakness, loss of ambulation (LoA), upper-limb function loss, progressive cardiac and respiratory problems, and premature death [1]. DMD is the most common form of muscular dystrophy with an incidence of from 1 in 3500 to 1 in 5000 live male births [2].

DMD is caused by mutations in the dystrophin (*DMD*) gene, which encodes the sarcolemmal protein, dystrophin. Around 60–70% of *DMD* gene mutations are large deletions, 5–15% are duplications, and 20% are point mutations, small deletions or insertions [3,4]. Dystrophin is expressed at the plasma membrane of all muscles, including cardiac, smooth, and skeletal muscle, as well as some neurons, and is known to connect the dystrophin-associated protein complex (DAPC) and the intracellular cytoskeleton c-actin [5]. Dystrophin maintains cytoskeletal stabilization; therefore, a lack of functional dystrophin causes membrane damage in skeletal muscle, which leads to progressive and irreversible muscle loss [6].

The earliest symptoms are difficulties with climbing stairs, gait disturbance and frequent falls; patients usually present with these symptoms before the age of 5 years [7]. Most patients become wheelchair dependent by the age of 13 and need assisted ventilation at around 20 years of age [1]. Patients can also develop cognitive impairment and learning and behavioral problems [8]. Recent meta-analyses have estimated median life expectancy to be between 28.1 and 29.9 years in current DMD populations [9,10]. There has been an improvement in survival over the years; a retrospective study from France revealed that, while the life expectancy of patients born before 1970 was 25.77 years, it increased to 40.95 years for those born after 1970 [11].

No cure exists for DMD and many therapeutics are being investigated in clinical trials, mainly aiming for prolonging ambulation, but also improving the impact of other aspects of DMD. The current standard of care includes rehabilitation, initiation of corticosteroid (CS), prevention and management of respiratory and cardiac complications, and psychosocial support [12,13,14]. Early initiation of CS treatment is strongly recommended, especially before physical decline [12]. Physical therapy is helpful to prolong ambulation, prevent contractures and deformities and keep functionality in all areas [12,13].

More recently, ataluren, a read-through drug, was shown to delay pulmonary and ambulatory decline compared with propensity-score-matched natural history controls [15]. Ataluren is indicated for the treatment of Duchenne muscular dystrophy resulting from a nonsense mutation in the dystrophin gene in ambulatory patients aged 2 years and older in the European Member States and Iceland, Liechtenstein, Norway, Great Britain, Northern Ireland, Kazakhstan, Israel, Belarus, Russia, Brazil, Chile, Macedonia, Uruguay and Serbia and aged 5 years and older in the KSA and Ukraine (under special state registration). In Brazil, the indication is specific to male pediatric patients. The presence of a nonsense mutation in the dystrophin gene should be determined by genetic testing [16]. Novel therapies such as gene therapy are also emerging to treat underlying disease mechanisms in DMD and are expected to be game changers for future DMD management [17]. Hence, the first gene therapy product for ambulatory DMD patients 4 through 5 years of age was recently approved by the United States Food and Drug Administration (FDA) [18].

A recent expert report discussing the current management of DMD in the Middle East and North Africa (MENA) highlighted that clinical practice across the region was variable and disease awareness was low [19]. Furthermore, there is limited information about disease characteristics and genetic profiles from the region [20,21,22,23]. Therefore, Saudi Arabian pediatric neurologists led by Dr. Al Saman, Primary Investigator, have initiated an ambispective, observational, and multicenter study of patients aged 1–14 years with genetically confirmed DMD to review the current practice and natural history of DMD and publish the preliminary findings from 177 patients diagnosed between 2014 and 2021 [24]. Here, we present final results of this ambispective cohort study to provide an up-to-date overview of genetic mutations, demographics, clinical characteristics, and initial management plans of DMD patients in order to evaluate the standard of care and its evolution over time in Saudi Arabia.

## 2. Method

### 2.1. Study Design

This was an ambispective observational multicenter study conducted in nine study sites across the KSA that performed DMD genetic testing as per their routine clinical practice.

**Figure 1:**
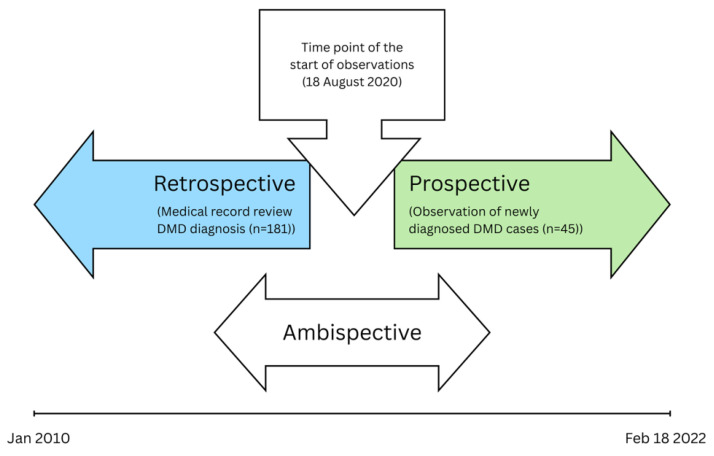
Study diagram.

The study consisted of retrospective and prospective arms. Patients were either in the prospective or retrospective arm of the study. None of the patients were enrolled both retrospectively and prospectively (Figure 1). The retrospective part of the study involved a retrospective review of medical records from January 2010 to 18 August 2020 to identify all cases of DMD diagnosed between January 2010 and August 2020 (study start date). The prospective part of the study included observation of detected cases of DMD within the 18 months after the study start date (from 18 August 2020 to 18 February 2022). The variables of interest evaluated for the study were: demographics, genetic mutations, clinical characteristics, and initial management. The relationship between the age at diagnosis, initial management plan (standard of care), and age at initiation of treatment on disease outcomes (wheelchair-bound status and age at LoA) were also evaluated.

### 2.2. Study Population

For retrospective patients, eligibility criteria were assessed during the screening process based on medical records, including genetic test results. Patients who did not satisfy all inclusion criteria or met any exclusion criteria were considered screen failures. For prospective patients, eligibility criteria also included written informed consent provided by the patient’s legal representative, as well as written informed assent from patients aged from 12 to 14 years, where applicable.

Patients were eligible for inclusion if they were Saudi citizens, male, and aged between 1 and 14 years with a genetically confirmed diagnosis of Duchenne muscular dystrophy (DMD). For the retrospective cohort, patients were identified through medical records with a confirmed diagnosis of DMD between January 2010 and 18 August 2020. For the prospective cohort, eligible patients were those diagnosed between 18 August 2020 and 18 February 2022. For prospective patients, informed consent from the legal guardian and informed assent from patients aged from 12 to 14 years were required prior to enrollment, whereas retrospective patients were exempt from these requirements.

Patients were excluded if they were not Saudi citizens or if prospective patients aged from 12 to 14 years did not provide the required informed assent.

### 2.3. Statistics

This study was designed as a descriptive observational study and, therefore, no hypothesis-testing statistical analyses were planned. Summary statistics were used to describe the collected variables. Numerical variables were summarized using: mean, standard deviation, median, range, and interquartile range. Categorical variables were summarized using frequency distributions. IBM SPSS software was used for the statistical analysis. Missing data were not imputed. Missing data for categorical variables were counted and presented as a separate “missing” category if they amounted to more than 10%.

### 2.4. Ethics Statement

All participating sites received Institutional Review Board (IRB) approvals for this ambispective observational multicenter study, which included both retrospective and prospective components. The approved protocol specified that both retrospective and prospective data would be collected as part of routine clinical practice. All patients of the prospective cohort signed an informed consent form to participate in the research according to Saudi Arabian law. An institutional board reviewed and agreed with the study protocol and the informed consent form. Patients and their legal representatives were provided with full and adequate verbal and written information regarding the objectives and procedures of the study. An informed consent document, approved by an Institutional Review Board, was provided to each prospective patient/patient’s legal representative. Where applicable, prospective patients from 7 to 14 years old also signed an age-appropriate assent form. By protocol amendment, verbal consent was permitted during the prospective period to accommodate coronavirus disease 2019 (COVID-19) restrictions.

## 3. Results

Of the 253 patients identified, 226 patients met the eligibility criteria and were included in the study. A total of 181 patients were enrolled in the retrospective part; 45 patients were enrolled in the prospective part.

### 3.1. Demographics

The median age of patients was 7.0 (interquartile range [IQR]: 4.8–8.5). In total, 167/226 (73.9%) were between 5 and 14 years of age, 57/226 (25.2%) were between 2 and 5 years of age and only two patients (0.9%) were under the age of 2.

Region of origin data were available for 225/226 patients and current region data were available for 218/226 patients. In total, 45/226 patients (20%) were originally from Makkah and 42/226 (18.7%) were from Riyadh. Almost half of the patients (48.6%) were currently located in these two cities too (Supplementary Material Table S9).

In total, 23/226 patients (10.2%) were diagnosed as having a comorbid disease, whereas 18/226 (8.0%) had only one and 5/226 (2.2%) had two other medical diagnoses. Regarding comorbidities, the most common was bronchial asthma (four patients [1.8%]) followed by attention deficit hyperactivity disorder (ADHD), autism and epilepsy, each reported in two patients.

### 3.2. Dystrophin Mutations

A summary of the mutation distribution is shown in Table 1. The most common type of genetic mutation among the 226 enrolled patients was large deletions (134 patients, 59.3%). More than 30% of patients had small sequence variants and nonsense mutations were detected in 39 patients (28.2%). Novel hemizygous gross segmental duplication (EX8_20dup & EX61_67dup) has been identified in one of the patients.

### 3.3. Clinical Characteristics

Clinical characteristics of patients are summarized in Table 2. The median age of first symptom was 2.7 years (IQR: 2.0–4.6 years) and the median age at diagnosis was 7.0 years (IQR: 4.8–8.5 years). Although 80% of patients were younger than 5 years at the time of first symptoms, less than 30% of patients had received a DMD diagnosis before the age of 5 years. In terms of gross motor development, approximately one third of patients were not walking by the age of 18 months.

In total, 195/226 patients (86.3%) had data available for walking characteristics. Among these patients, the most common observed characteristic was difficulty with walking (87.7%) and waddling gait (41%). Across all 226 patients, 64 (28.3%) were fully wheelchair-bound at the time of study entry, particularly the ones in the retrospective arm of the study. The median age at LoA was 10.6 years (IQR: 9.1–11.6 years). Among the ambulatory patients, 55/127 (43.3%) were able to run and 75/127 (59.1%) were able to climb.

The most frequent clinical finding was calf enlargement (86.7%) followed by lumbar lordosis (40.9%), lumbar scoliosis (32.5%) and Trendelenburg gait (29.1%). Sleep apnea and DMD-associated cardiomyopathy were only reported in patients involved in the retrospective arm, most likely due to the longer follow-up. In total, 187/226 patients had available data for intellectual capacity and only approximately one fourth of the patients were reported to have intellectual impairment.

While pulmonary functions were tested in all prospective-arm patients, data were available for only 10% of the retrospective-arm patients. The median FEV1/FVC levels were 98.5% (IQR: 84.0–113.0%) in prospectively enrolled patients and 78% (IQR: 68.0–116.0) in the retrospectively enrolled ones. Among the patients with available laboratory data, serum creatine kinase (CK), alanine aminotransferase (ALT), and aspartate aminotransferase (AST) were elevated, which is consistent with DMD disease.

**Figure 2:**
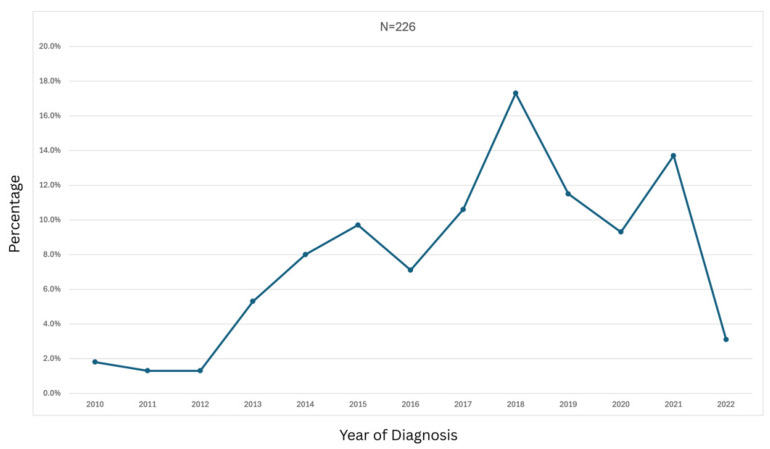
Diagnosis rate of DMD patients over the years (*n* = 226). The apparent decrease in diagnoses in 2022 is due to the study observation period ending in February 2022; therefore, data for that year represent only the first two months of observation.

The rates of symptom identification and DMD diagnosis (Figure 2) increased over time and generally peaked between 2014 and 2018, reflecting increased awareness of DMD on the part of healthcare providers and parents and availability of genetic testing. Declines seen after 2019 are likely caused by plateauing of the data and the effect of COVID-19 restrictions on hospital visits. While two third of patients (61.5%) were referred from another center, only 20% of patients were diagnosed at the referral center. Almost 80% of patients were diagnosed at their current treatment site.

### 3.4. Initial Management

The initial management plan for DMD patients in the KSA mostly involved medication (75.6%) and physical therapy (71.0%). The most frequently prescribed initial medications were vitamin D (82%) and steroids (62.3%). Ataluren, which is indicated for the treatment of nonsense mutation Duchenne muscular dystrophy (nmDMD), was included in the initial management plan for 6 out of 226 patients (one of them had ataluren alone and five of them had ataluren with CS treatment).

The median age of CS initiation was 7.1 years (IQR: 5.7–8.7). Although few patients in the prospective part of the study have data regarding the age at initiation of steroid use, the results suggest that there is an increasing trend to start CS treatment earlier. Among the 111 patients who received CS, 92 had prednisolone, seven had prednisone and two patients received deflazacort treatment (Table 3).

### 3.5. Efficacy Evaluation

Patients who received CS treatment were less likely to be fully wheelchair-bound compared to the non-treated ones (27% versus 37%, respectively). And, although only five patients received ataluren with CS, the data suggest that the combination may be associated with better outcomes than standard care alone (Table 4).

While the median age at LoA was 9.8 years (IQR: 8.0–11.4 years) in the non-treated patients, it was 10.1 years (IQR: 9.3–11.2 years) in the steroid-only group and 10.8 years (10.8, 10.8) in the combined ataluren and steroid treatment group (Table 4).

Patients who were diagnosed before the age of 5 years were much more likely to retain ambulation than those who were diagnosed after the age of 5 years. While only 6 of 59 patients (10.2%) diagnosed before the age of 5 years had lost ambulation, 58 of 167 patients (34.7%) diagnosed after the age of 5 years became wheelchair-bound.

Age of treatment initiation was known for 90/226 patients and outcomes were significantly better for patients who started treatment before the age of 7 years. Approximately half of the 47 patients who initiated treatment after the age of 7 years (23 patients, 48.9%) were fully wheelchair-bound, whereas only 4/43 patients (9.3%) who received treatment before the age of 7 years lost ambulation (Table 5).

## 4. Discussion

To the best of our knowledge, this is the largest study of DMD patients in the KSA elucidating demographical, clinical, diagnostic and treatment characteristics of 226 patients diagnosed between 2014 and 2022. Furthermore, this is the first study to provide a holistic view of the heterogeneity in disease management and the response to different therapeutic modalities within the Saudi population.

According to the Saudi statistical report, Riyadh and Makkah had the highest population among other regions. The highest number of cases were from the Riyadh and Makkah provinces, establishing an east–west “axis” across the center of the country, and almost half of the patients are in these two provinces. The geographic distribution of patients observed in this study should be interpreted with caution. The apparent concentration of cases in major urban centers, particularly Riyadh and Makkah, may reflect referral patterns rather than true regional differences in disease prevalence. These cities host several tertiary referral centers with specialized pediatric neurology and genetic diagnostic services, which serve as major hubs for the diagnosis and management of neuromuscular disorders, including Duchenne muscular dystrophy. As a result, patients from other regions of the Kingdom are frequently referred to these centers for specialized evaluation and genetic confirmation. Consequently, the geographic distribution observed in this cohort likely reflects the centralization of specialized care rather than underlying regional variation in disease incidence.

Thousands of different mutations have been found in patients with DMD. Most of the reports illustrate that the spectrum of *DMD* gene mutations involve 79% of the patients reportedly having structural variants, of which 68% were large deletions and 11% were large duplications [25]. The remaining 20–25% of patients carried small sequence variants and almost half of them were nonsense mutations [17,25,26]. Small deletions, small insertions, and splice site mutations account for 5%, 2%, and 3% of total mutations, respectively [17,25,27]. In our cohort, while structural variants (deletions and duplications) comprise 70% of total mutations, the rate of small sequence variants was higher than previous reports (29.6% of total mutations). Nonsense mutations were also more frequent in Saudi patients compared to earlier studies, as they accounted for 17.3% of the mutations in the total patient population.

Motor symptoms such as difficulties in running, jumping, rising from the floor (Gower’s sign), struggling to hop or climb stairs, frequent falls, and abnormal and/or waddling gait are the most typical signs reported in DMD patients [28]. However, delays in early developmental milestones can also be important to recognize DMD early. The mean age of independent walking was 16.35 months in the DMD patients compared with 12.26 months in the control group [29]. In our cohort, almost 30% of patients could not achieve independent walking by the age of 18 months. While 38–45% of DMD patients are reported to have speech and language delay in previous reports, 65% of patients in the Saudi cohort experienced a delay in speech [30,31].

Mean age at symptom onset was 2.9 years in Austria and Germany, 2.6 years in Italy, 2.7 years in the UK and the USA and 2.85 years in Australia [31,32,33,34]. Mean age at symptom onset was slightly later in our cohort and was reported as 3.6 years. In Austria, DMD was diagnosed at a mean age of 3.7 years, 7 months after symptom onset. Age of diagnosis of DMD in Austria, Germany, Italy, Australia, the UK, and the USA was reported at 3.7, 3.4, 3.4, 3.8, 4.3 and 4.9 years of age, respectively [31,32,33,34,35]. A cross-European study revealed a mean time to diagnosis of about 15 months from the first symptom onset, with a mean age of diagnosis at 4.3 years of age [36]. Our study revealed that, besides detecting symptoms later than western countries, there is also a significant delay between symptom onset and diagnosis in the KSA. Although symptoms may be subtle at the earlier stages of the disease, this delay can also be a result of a lack of awareness in considering the diagnosis of DMD.

Early diagnosis means early access to treatments and specialist care; therefore, developmental screening tools such as milestone checklists are important for diagnosis. And, checking creatine kinase levels early in the evaluation of boys with unexplained developmental delay is strongly recommended [37]. In the case of elevated CK levels, patients should be referred to pediatric neurologists. While 75% of the patients were referred to a specialized center for diagnosis in Austria, the referral rate was more than 60% in the KSA [34].

Previous studies have reported several conditions affecting cognition, behavior, and gastrointestinal and genitourinary functioning that could be considered secondary to the diagnosis of DMD [38]. These secondary conditions involve additional physical or mental problems that are related to the underlying disease. In our cohort, almost one tenth of DMD patients in our cohort were diagnosed with either one or two of these secondary conditions. Bronchial asthma was the most common secondary condition in our patients with a rate of 1.8%. However, asthma was recorded in 10.4% of DMD patients registered in the Muscular Dystrophy Surveillance, Tracking, and Research Network (MD STARnet) [38].

Cognitive and neurobehavioral problems were frequently reported in DMD patients. A recent report from Canada revealed that a full-scale IQ of <70 was seen in 27%, learning disability in 44%, intellectual disability (ID) in 19%, ADHD in 32%, autism spectrum disorders in 15%, and anxiety in 27% of DMD patients [39]. Similarly, a multicentric study from Italy revealed that the incidence of ID and ADHD was 24.6% and 32%, respectively [40]. In our cohort, almost one fourth of patients were reported to have ID, whereas 16.3% had speech delay, 13.2% had learning difficulties, 3% had low IQ and 1.3% had autism-like behaviors. ADHD was only recorded in 4/226 patients in our study.

In the KSA, 62.3% patients were treated with CS, which was very similar to the rate of 65.2% in Europe. However, CS therapy is started later in the KSA compared to European countries; the mean age at the start of therapy was 7.4 and 6.1 years, respectively [36]. CS treatment is associated with prolonged ambulation. In our cohort, patients who received CS treatment were less likely to be fully wheelchair-bound (27% versus 37%). Among studies of CS-treated patients, the mean age at LOA ranged from 9.5 years to 12.5 years of age [41,42]. The early start and long-term usage of CS were associated with better clinical outcomes including prolonged ambulation and delayed development of cardiac and respiratory involvement [43]. The earliest mean age at LOA was 9.5 years in patients with ≤3 years of CS treatment compared with 12.3 years in those with >3 years of CS use (MD STARnet) [41]. As the age of treatment onset was relatively late in our cohort, the age at LOA was 10.3 and 9.6 years in CS-treated and non-treated patients, respectively. However, early start of CS was associated with better motor function in our cohort too. While approximately half of the patients who started CS treatment after the age of 7 years became fully wheelchair-bound, less than 10% of patients lost ambulation in the group that started CS before the age of 7 years.

Although most of the patients in the Saudi cohort received prednisolone/prednisone treatment, a recent study revealed that deflazacort was associated with better outcomes, especially among older/more progressed patients [44,45]. Ataluren promotes the production of full-length dystrophin via the read-through of an in-frame premature stop codon. It was shown to slow down motor decline and respiratory disease progression in nmDMD compared to CS alone [15,46,47]. Although only five patients received ataluren treatment with CS in our cohort, 4/5 (80%) of them preserved their ambulation and independent living. It should be noted that the results for the combined ataluren + steroid group (*n* = 6) are preliminary and not generalizable.

## 5. Conclusions

This study provides one of the largest multicenter descriptions of the genetic and clinical characteristics of Duchenne muscular dystrophy (DMD) patients in the Kingdom of Saudi Arabia. The findings highlight important gaps in the diagnostic pathway, particularly the delay between the onset of symptoms and confirmed diagnosis. These results underscore the need to strengthen early recognition of DMD in routine clinical practice through improved awareness among primary care physicians, pediatricians, and allied healthcare professionals. Earlier referral for genetic testing and specialist evaluation could facilitate more timely diagnosis and earlier initiation of standard-of-care interventions, which may help delay disease progression and improve long-term outcomes.

At a broader healthcare system level, the findings support the development of structured awareness initiatives and educational programs aimed at improving the recognition of early neuromuscular symptoms in children. The implementation of standardized referral pathways and consideration of targeted screening strategies in high-risk populations may further reduce diagnostic delays. Strengthening national or regional patient registries would also help improve the understanding of disease epidemiology, mutation patterns, and treatment practices in the region.

## 6. Future Directions

Future research should prioritize prospective longitudinal studies with standardized genetic and clinical data collection to better characterize disease progression, treatment response, and long-term outcomes in the regional DMD population. Improved genetic reporting, including exon-level mutation characterization and standardized variant annotation, would facilitate the evaluation of mutation-specific therapies such as exon-skipping approaches. In addition, more comprehensive and standardized documentation of comorbidities and neurodevelopmental conditions would provide a clearer understanding of the broader disease burden associated with DMD. These efforts will be important for informing evidence-based clinical management strategies and guiding healthcare planning for DMD in Saudi Arabia and the wider region.

## 7. Limitations

This study has several limitations that should be considered when interpreting the findings. First, genetic testing was not performed as part of the study protocol; instead, the study relied on genetic test results documented in patient medical records as part of routine clinical care. Because the study included retrospective cases diagnosed across multiple institutions over several years, genetic testing was conducted at different laboratories and the specific assay methods used were not consistently documented. In addition, centralized genetic analysis or standardized variant classification was not performed. Consequently, detailed information regarding testing platforms (e.g., aCGH, targeted sequencing, or next-generation sequencing panels), laboratories performing the analyses, and variant classification according to ACMG criteria was not consistently available.

The available dataset primarily included mutation categories (e.g., deletions, duplications, and small sequence variants) rather than detailed mutation-level annotations such as genomic coordinates, HGVSc/HGVSp descriptions, or ClinVar classifications. As a result, exon-level characterization of structural variants and the generation of a mutation-level catalog were not possible. Similarly, detailed molecular information on structural variants, including nucleotide length, exon coordinates, or genomic breakpoints, was not consistently available and, therefore, the size of structural variants could not be reported.

Another limitation relates to the availability of clinical data extracted from medical records. Although comorbid conditions were reported in 10.2% of patients, the specific types of comorbidities were not systematically captured. Consequently, clinically relevant complications commonly associated with DMD, such as gastrointestinal complications or fracture frequency, could not be analyzed in detail. In addition, the relatively low number of reported ADHD diagnoses may reflect under-diagnosis or under-reporting rather than a true difference in prevalence, as neurodevelopmental conditions may not have been systematically evaluated or documented in all patients.

The observational and partly retrospective design of the study also introduces limitations. Data were collected at a single time point for each patient, and no longitudinal follow-up was conducted. Consequently, disease progression over time could not be assessed, and the evaluation of treatment effects for corticosteroids or ataluren reflects cross-sectional observations rather than longitudinal outcomes. Therefore, treatment effects cannot be interpreted as causal and may be subject to selection bias.

Finally, there was an imbalance between the retrospective (*n* = 181) and prospective (*n* = 45) cohorts, reflecting the study design in which cases were retrospectively identified over a longer period (2010–2020), while prospective observation was limited to an 18-month period. This difference could not be modified retrospectively and, together with missing data in some variables, particularly in the retrospective arm where assessments such as pulmonary function tests were available only for a subset of patients, may limit the statistical power of comparative analyses. In addition, the cohort included patients across a wide age range and at different stages of disease progression, which may introduce further heterogeneity in the clinical data.

## Figures and Tables

**Table 1:** Distribution of identified mutations in DMD population.

Type of Identified Mutation	Retrospective (*N* = 181)			Prospective (*N* = 45)			Overall (*N* = 226)		
	*n*	%	95% Confidence Interval	*n*	%	95% Confidence Interval	*n*	%	95% Confidence Interval
Large deletion	104	57.5%	49.9–64.8%	30	66.7%	51.0–80.0%	134	59.3%	52.6–65.8%
Large duplication	23	12.7%	8.2–18.5%	2	4.4%	0.5–15.1%	25	11.1%	7.3–15.9%
Small sequence variants *	54	29.8%	23.3–37.1%	13	28.9%	16.4–44.3%	67	29.6%	23.8–36.1%
Nonsense mutations	35	19.3%	13.9–25.9%	4	8.9%	2.5–21.2%	39	17.3%	12.6–22.8%
Deletion/insertion mutations	16	8.8%	5.1–14.0%	9	20.0%	9.6–34.6%	25	11.1%	7.3–15.9%
Splicing mutations	3	1.7%	0.3–4.8%	0	0.0%	0.0–7.9%	3	1.3%	0.3–3.8%
Other mutations	3	1.7%	0.3–4.8%	0	0.0%	0.0–7.9%	3	1.3%	0.3–3.8%
Total	181			45			226		
Patients with two or more small sequence variants	3	1.7%	0.3–4.8%	0	0.0%	0.0–7.9%	3	1.3%	0.3–3.8%

* three patients had two small sequence variants at the same time.

**Table 2:** Clinical characteristics of 226 DMD patients in the KSA.

Variable	Retrospective Patients (*N* = 181)	Prospective Patients (*N* = 45)	Total (*N* = 226)
**Age at diagnosis (years), *n* (%)**	***N*** **= 181**	***N*** **= 45**	***N*** **= 226**
<2	2 (1.1)	0	2 (0.9)
≥2 to <5	44 (24.3)	13 (28.9)	57 (25.2)
≥5 to 14	135 (74.6)	32 (71.1)	167 (73.9)
**Age at diagnosis (years)**	***N*** **= 181**	***N*** **= 45**	***N*** **= 226**
Mean (SD)	7.0 (2.8)	6.7 (2.5)	6.9 (2.7)
Median (Q1, Q3)	6.9 (4.9, 8.6)	7.2 (4.5, 8.2)	7.0 (4.8, 8.5)
Min, Max	1.3, 13.8	2.3, 12.9	1.3, 13.8
**Age at first sign and symptoms (years), *n* (%)**	***N*** **= 97**	***N*** **= 45**	***N*** **= 142**
<2	21 (21.6)	14 (31.1)	35 (24.6)
≥2 to <5	54 (55.7)	25 (55.6)	79 (55.6)
≥5 to 14	22 (22.7)	6 (13.3)	28 (19.7)
**Age at first sign and symptoms (years)**	***N*** **= 97**	***N*** **= 45**	***N*** **= 142**
Mean (SD)	3.8 (2.3)	3.2 (2.3)	3.6 (2.3)
Median (Q1, Q3)	2.9 (2.0, 4.9)	2.5 (1.9, 3.5)	2.7 (2.0, 4.6)
Min, Max	0.4, 10.3	0.6, 10.6	0.4, 10.6
**Walking characteristics, *n* (%)**	***N*** **= 181**	***N*** **= 45**	***N*** **= 226**
Difficulty on walking	131 (87.3)	40 (88.9)	171 (87.7)
Walks on their toes	28 (18.7)	16 (35.6)	44 (22.6)
Waddles	54 (36.0)	26 (57.8)	80 (41.0)
Others	9 (6.0)	2 (4.4)	11 (5.6)
**Patient fully wheelchair-bound, n (%)**	***N*** **= 181**	***N*** **= 45**	***N*** **= 226**
Yes	60 (33.1)	4 (8.9)	64 (28.3)
No	86 (47.5)	41 (91.1)	127 (56.2)
Not available	35 (19.3)	0	35 (15.5)
Missing	0	0	0
**Age at fully wheelchair-bound (years)**	***N*** **= 27**	***N*** **= 3**	***N*** **= 30**
Mean (SD)	10.5 (2.4)	9.7 (1.6)	10.4 (2.3)
Median (Q1, Q3)	10.7 (9.3, 12.1)	9.0 (8.6, 11.6)	10.6 (9.1, 11.6)
Min, Max	5.0, 15.5	8.6, 11.6	5.0, 15.5
**Clinical findings ^a^, *n* (%)**	***N*** **= 163**	***N*** **= 40**	***N*** **= 203**
Enlargement of the calves	137 (84.0)	39 (97.5)	176 (86.7)
Lumbar lordosis	65 (39.9)	18 (45.0)	83 (40.9)
Lumbar scoliosis	51 (31.3)	15 (37.5)	66 (32.5)
Trendelenburg gait	40 (24.5)	19 (47.5)	59 (29.1)
Sleep apnea	1 (0.6)	0	1 (0.5)
DMD-associated cardiomyopathy	9 (5.5)	0	9 (4.4)
Other	7 (4.3)	2 (5.0)	9 (4.4)
**Intellectual impairment, *n* (%)**			
Yes	46 (25.4)	11 (24.4)	57 (25.2)
No	97 (53.6)	33 (73.3)	130 (57.5)
Not available	38 (21.0)	1 (2.2)	39 (17.3)
Missing	0	0	0
**Pulmonary function tests, *n* (%)**			
Yes	19 (10.5)	2 (4.4)	21 (9.3)
No	97 (53.6)	43 (95.6)	140 (61.9)
Not available	65 (35.9)	0	65 (28.8)
**FEV1 (L)**			
Mean (SD)	1.0 (0.0)	1.5 (0.7)	1.0 (0.2)
Median	1.0	1.5	1.0
Min, Max	1.0, 1.0	1.0, 2.0	1.0, 2.0
**FVC (L)**			
Mean (SD)	1.1 (0.2)	1.5 (0.7)	1.1 (0.3)
Median	1.0	1.5	1.0
Min, Max	1.0, 2.0	1.0, 2.0	1.0, 2.0
**FEV1/FVC (%)**			
Mean (SD)	84.1 (16.7)	98.5 (20.5)	85.5 (17.1)
Median	78	98.5	79.0
Min, Max	68.0, 116.0	84.0, 113.0	68.0, 116.0
**Predicted FVC (L)**			
Mean (SD)	4.5 (14.7)	36.0 (49.5)	7.5 (20.1)
Median	1.0	36	1.0
Min, Max	1.0, 65.0	1.0, 71.0	1.0, 71.0
**ALT (U/L)**	***N*** **= 115**	***N*** **= 21**	***N*** **= 136**
Mean (SD)	348.6 (186.6)	334.1 (165.4)	346.4 (182.9)
Median	332.0	327.0	331.0
Min, Max	36.0, 1181.0	81.0, 746.0	36.0, 1181.0
**AST (U/L)**	***N*** **= 99**	***N*** **= 18**	***N*** **= 117**
Mean (SD)	246.7 (154.3)	244.1 (158.5)	246.3 (154.3)
Median	211.0	189.5	210.0
Min, Max	48.0, 967.0	45.0, 623.0	45.0, 967.0
**CK (U/L)**	***N*** **= 147**	***N*** **= 27**	***N*** **= 174**
Mean (SD)	12,585.3 (7625.4)	12,336.6 (7795.4)	12,546.7 (7629.8)
Median	11,017.0	11,350.0	11,051.0
Min, Max	759.0, 50,953.0	1100.0, 29,535.0	759.0, 50,953.0

^a^: Percentages did not add up to 100, as a patient might have multiple present clinical characteristics.

**Table 3:** Treatment characteristics of DMD patients.

Variable	Retrospective Patients (*N* = 181)	Prospective Patients (*N* = 45)	Total (*N* = 226)
**Age at CS initiation (years)**	***N*** **= 78**	***N*** **= 11**	***N*** **= 89**
Mean (SD)	7.5 (2.4)	6.7 (1.9)	7.4 (2.3)
Median (Q1, Q3)	7.3 (5.9, 8.7)	6.6 (4.8, 8.2)	7.1 (5.7, 8.7)
Min, Max	2.6, 14.2	4.2, 10.2	2.6, 14.2
**Age group (years) at CS initiation, *n* (%)**			
≥2 to <5	13 (16.7)	3 (27.3)	16 (18.0)
≥5 to <7	22 (28.2)	4 (36.4)	26 (29.2)
≥7	43 (55.1)	4 (36.4)	47 (52.8)
**Age at ataluren initiation (years)**	***N*** **= 6**		***N*** **= 6**
Mean (SD)	7.2 (2.4)	-	7.2 (2.4)
Median (Q1, Q3)	7.5 (5.7, 8.8)	-	7.5 (5.7, 8.8)
Min, Max	2.9, 9.9	-	2.9, 9.9
**Age group (years) at ataluren initiation, *n* (%)**			
<7	2 (33.3)	-	2 (33.3)
≥7	4 (66.7)	-	4 (66.7)

**Table 4:** Effects of CS and ataluren on the ambulation status of DMD patients.

		CS Only (*n* = 104)		CS + Ataluren (*n* = 5)		No CS or Ataluren (*n* = 54)	
		*n*	%	*n*	%	*n*	%
**Is the patient fully wheelchair-bound?**	Yes	29	27.9%	1	20.0%	20	37.0%
	No	71	68.3%	4	80.0%	29	53.7%
	Not Available	4	3.8%	0	0.0%	5	9.3%
Age at loss of ambulation *	***n*** **Mean/±SD**	15 10.3 ± 2.3	10.8 ± 0	1 9.6 ± 2.7		8	
	**Median/(Q1–Q3)**	10.1 (9.3, 11.2)	10.8 (10.8, 10.8)	9.8 (8.0, 11.4)			
	**Min/Max**	4.9–15.5	10.8–10.8	5.0–13.6			
	***n*** **missing**	14		0		12	

* among those fully wheelchair-bound.

**Table 5:** Ambulation characteristics of the included patients according to age of treatment onset.

		Age at Start of Treatment					
		<7 years (*n* = 43)		>7 years (*n* = 47)		Total (*n* = 90)	
		***n***	**%**	***n***	**%**	***n***	**%**
Is the patient fully wheelchair-bound?	Yes	4	9.3%	23	48.9%	27	30.0%
	No	36	83.7%	23	48.9%	59	65.6%
	Not Available	3	7.0%	1	2.1%	4	4.4%
Is the patient able to run? *	Yes	17	47.2%	11	47.8%	28	47.5%
	No	10	27.8%	10	43.5%	20	33.9%
	Not Available	9	25.0%	2	8.7%	11	18.6%
	Yes	24	66.7%	16	69.6%	40	67.8%
Is the patient able to climb stairs? *	No	8	22.2%	6	26.1%	14	23.7%
	Not Available	4	11.1%	1	4.3%	5	8.5%

* Percentages for “Is the patient able to run?” and “Is the patient able to climb stairs?” were calculated among patients who were not fully wheelchair-bound.

## Data Availability

The data presented in this study are not publicly available due to patient confidentiality and institutional restrictions. Data may be available from the corresponding author upon reasonable request.

## Supplementary Materials

The following supporting information can be downloaded at: https://www.mdpi.com/article/10.3390/healthcare14070857/s1. Supplementary Material File: Clinical Study Report [7,12,19,21,22,23,24,26,36,48,49,50,51,52,53,54,55,56,57,58,59,60,61,62,63,64,65,66].
