# Supplementary material for: Genetic, Clinical, and Management Characteristics of Duchenne Muscular Dystrophy in Saudi Arabia
Source: Healthcare (Basel). 2026 Mar 27;14(7):857. doi: 10.3390/healthcare14070857 (PMC13073469; doi:10.3390/healthcare14070857)
Supplement: Supplementary file 1 [file healthcare-14-00857-s001.zip › healthcare-4127564-supplementary.pdf]

PTC-GD-MA-405

AMBISPECTIVE OBSERVATIONAL MULTICENTER STUDY TO DESCRIBE THE GENETIC MUTATIONS AND CLINICAL CHARACTERISTICS OF DUCHENNE MUSCULAR DYSTROPHY DISEASE IN KINGDOM OF SAUDI ARABIA

Indication studied:	*Duchenne Muscular Dystrophy*
Release date of report:	*31 October 2023*

Company/Sponsor signatory:	*Christian Werner*
	+49 (0) 172 925 39 03

This trial was conducted in accordance with the ethical principles of Good Clinical Practice, according to the ICH Harmonized Tripartite Guideline.

Sponsor's Responsible Medical Officer: Dr. med. Christian Werner
Executive Director Global Medical Affairs
Global DMD Lead
PTC Therapeutics Germany GmbH.

The eSignature Page is located on the last page.

Notice of Proprietary Information: This document contains confidential information owned by or in the possession/control of PTC Therapeutics, Inc. Except as may otherwise be permitted in writing, by accepting or reviewing these materials, you agree that this information should not be disclosed to others (except where required by applicable law) and should not be used for unauthorized purposes. In the event of an actual or suspected breach of this obligation, PTC Therapeutics, Inc. should be notified promptly.

2. SIGNATURE OF SPONSOR'S RESPONSIBLE MEDICAL OFFICER

AMBISPECTIVE OBSERVATIONAL MULTICENTER STUDY TO DESCRIBE THE GENETIC MUTATIONS AND CLINICAL CHARACTERISTICS OF DUCHENNE MUSCULAR DYSTROPHY DISEASE IN KINGDOM OF SAUDI ARABIA

I have read this report and confirm that to the best of my knowledge it accurately describes the conduct and results of the study.

______________________________	______________
**Dr. med. Christian Werner**<br>Executive Director Global Medical Affairs, Global DMD Lead<br>PTC Therapeutics Germany GmbH	**Date**

3. TABLE OF CONTENTS FOR THE INDIVIDUAL CLINICAL STUDY REPORT

TABLE OF CONTENTS

1. TITLE PAGE 1
2. SIGNATURE OF SPONSOR'S RESPONSIBLE MEDICAL OFFICER 2
3. TABLE OF CONTENTS FOR THE INDIVIDUAL CLINICAL STUDY REPORT 3
TABLE OF CONTENTS 3
LIST OF TABLES 5
LIST OF FIGURES 5
4. LIST OF ABBREVIATIONS AND DEFINITIONS OF TERMS 6
5. ETHICS 7
6. INVESTIGATORS AND STUDY ADMINISTRATIVE STRUCTURE 7
7. INTRODUCTION 8
7.1. Background 8
7.2. Study Rationale 9
8. STUDY OBJECTIVES 10
9. INVESTIGATIONAL PLAN 10
9.1. Overall Study Design and Plan: Description 10
9.1.1. Retrospective Part 11
9.1.2. Prospective Part 11
9.2. Discussion of Study Design, Including the Choice of Control Groups 12
9.3. Selection of Study Population 12
9.3.1. Inclusion Criteria 12
9.3.2. Exclusion Criteria 12
9.3.3. Patient Withdrawal 12
9.4. Treatments 13
9.5. Efficacy Variables and Schedule of Assessment 13
9.5.1. Schedule of Assessments 13
9.5.2. Primary Endpoint 13
9.5.3. Secondary Endpoints 13
9.6. Data Quality Assurance 15

9.7. Statistical Methods Planned in the Protocol and Determination of Sample Size 15
9.7.1. Statistical and Analytical Plans 15
9.7.1.1. Baseline Descriptive Statistics....16
9.7.1.2. Analysis of Primary and Secondary Endpoints 16
9.7.1.3. Safety Analyses 16
9.7.1.4. Planned Interim Analyses 16
9.7.2. Determination of Sample Size 16
9.8. Changes in the Conduct of the Study or Planned Analyses....16
10. STUDY PATIENTS 17
10.1. Disposition of Patients 17
10.2. Demographics and Other Baseline Characteristics....17
10.3. Clinical Characteristics 22
10.4. Initial Management Plan....24
11. EFFICACY EVALUATION 26
11.1. Effect of Medication on Physical Function 27
11.2. Effect of Age at Diagnosis and Intervention on Physical Function....28
11.2.1. Age at Diagnosis....28
11.2.2. Age at Start of Active Treatment....28
12. SAFETY EVALUATION 29
13. DISCUSSION AND OVERALL CONCLUSIONS 29
14. SUPPORTING TABLES....31
15. REFERENCE LIST 46

LIST OF TABLES

Table 1: Abbreviations and Specialized Terms 6
Table 2: Study Centers and Principal Investigators 7
Table 3: Study Administrative Personnel 7
Table 4: Study Objectives and Endpoints 10
Table 5: Visit Schedule 13
Table 6: Sample Size Estimations Based on Mutation Prevalence 16
Table 7: Summary of Study PTC-GD-MA-405 Substantive Protocol Changes 17
Table 8: Patient Disposition/Patient Completion Summary 17
Table 9: Summary of Demographics 18
Table 10: Genetic Mutations 19
Table 11: Duchenne Muscular Dystrophy Characteristics 20
Table 12: Walking Characteristics 22
Table 13: Clinical Characteristics 23
Table 14: Initial Management Plan 25
Table 15: Summary of Physical Therapy Management by Site and Steroid Treatment 26
Table 16: Ambulation Characteristics of the Included Patients According to Types of Prescribed Active Medication 27
Table 17: Age at Fully Wheelchair Bound by Type of Treatment 28
Table 18: Ambulation Characteristics by Age at Diagnosis 28
Table 19: Ambulation Characteristics of the Included Patients According to Age at the Start of Treatment 29

LIST OF FIGURES

Figure 1: Study Design 11
Figure 2: Year of First Signs and Symptoms of DMD 21
Figure 3: Year of Diagnosis of DMD 21
Figure 4: Components of Initial Management Plan 24
Figure 5: Physical Therapy Management by Clinical Site and Steroid Treatment 26
Figure 6: Bar Chart of Ambulation Characteristics by Age at the Start of Treatment 29

4. LIST OF ABBREVIATIONS AND DEFINITIONS OF TERMS

The following abbreviations and specialized terms are used in this study report.

Table 1: Abbreviations and Specialized Terms

Abbreviation or Specialized Term	Explanation
ALT	Alanine aminotransferase
AST	Aspartate aminotransferase
CK	Creatine kinase
DMD	Duchenne muscular dystrophy
eCRF	Electronic Case Report Form
FEV1	Forced expiratory volume in one second
FVC	Forced vital capacity
IAF	Informed Assent Form
IEC	Institutional Ethics Committee
IQ	Intelligence quotient
KSA	Kingdom of Saudi Arabia
MENA	Middle East and North Africa
MLPA	Multiplex ligation-dependent probe amplification
MOE	Margin of error
nmDMD	Nonsense mutation Duchenne muscular dystrophy
NGS	Next-generation sequencing
PCR	Polymerase chain reaction
PTC	PTC Therapeutics

5. ETHICS

Patient informed consent was obtained only in the prospective part of the study. Patients and their legal representatives were provided with full and adequate verbal and written information regarding the objectives and procedures of the study. An informed consent document, approved by an Institutional Review Board, was provided to each prospective patient/patient's legal representative. The patient's/legal representative's signature on the informed consent form was to be obtained at the investigator site in the presence of the investigator or a qualified representative. Where applicable, prospective patients 12 to 14 years old also signed an age-appropriate assent form. Each prospective patient/legal representative was given a copy of the signed consent form. The original signed documents were retained by the investigator with the study records. By protocol amendment, verbal consent was permitted during the prospective period to accommodate coronavirus disease 2019 (COVID-19) restrictions.

6. INVESTIGATORS AND STUDY ADMINISTRATIVE STRUCTURE

This ambispective, observational study was sponsored by PTC Therapeutics International Limited and conducted at 9 centers in the Kingdom of Saudi Arabia (KSA) (Table 2). The personnel responsible for administrative aspects of the study are identified in Table 3.

Table 2: Study Centers and Principal Investigators

Site Number	Site Name	Investigator Name
KSA-01	King Fahad Medical City	Dr. Abdulaziz Alsaman
KSA-02	King Faisal Specialist Hospital and Research Center – Riyadh	Dr. Mohammad Almuhaiezie
KSA-03	King Fahad Specialist Hospital – Dammam	Dr. Fouad Alghamdi
KSA-04	King Khaled University Hospital	Dr. Fahad Bishiri
KSA-05	King Saud Medical City	Dr. Baleeg Ali
KSA-06	King Abdulaziz University Hospital	Dr. Ahmad Bamaga
KSA-07	King Abdulaziz Medical City – NGHA Jeddah	Dr. Nahla Alshiekh
KSA-08	King Faisal Specialist Hospital and Research Center – Jeddah	Dr. Osama Muzzfar
KSA-09	National Guard Health Affairs – Riyadh	Dr. Mohammad Almuqbil

Table 3: Study Administrative Personnel

Role/Function	Person Responsible
**Coordinating Investigator**	Abdulaziz S. Alsaman<br>King Fahad Medical City<br>Department of Pediatric Neurology<br>Riyadh, Saudi Arabia

Role/Function	Person Responsible
**PTC Clinical Lead/Medical Monitor**	Arzu Mulayim, MD<br>Sr. Director, Medical Affairs<br>Middle East and North Africa<br>PTC Therapeutics Switzerland GmbH<br>Turmstrasse 28, Tower 2<br>CH-6312 Steinhausen/Zug<br>Switzerland<br>Phone: +41-79-839-7776<br>Email: amulayim@ptcbio.com
**PTC Biostatistician**	Emelline Liu<br>Executive Director, Biostatistics<br>Real World Evidence and Medical Affairs<br>Email: eliu@ptcbio.com
**PTC Study Manager**	Elena Heider<br>Senior Manager, Registry Operations, Medical Affairs<br>PTC Therapeutics Switzerland GmbH<br>Turmstrasse 28, Tower 2<br>CH-6312 Steinhausen/Zug<br>Switzerland<br>Phone: +41-79-615-5504<br>Email: eheider@ptcbio.com
**Data Management**	Hamees Abdelalim<br>Clinical Data Manager I<br>4th Floor, Building 340, S 90 Street<br>Fifth Settlement<br>New Cairo, Egypt<br>Phone: +2-0127-105-7799<br>Email: habdelalim@ctifacts.com

7. INTRODUCTION

7.1. Background

Duchenne muscular dystrophy (DMD) is a clinically heterogeneous, severe, rare, progressive, X-linked, recessive, genetic neuromuscular childhood disease with an incidence of 1 in 3600 to 5000 male births regardless of race or ethnicity (Emery 1991, Bushby 2010b, Ellis 2013). DMD is caused by mutations in the dystrophin gene, which comprises 79 exons that produce dystrophin-called membrane protein, and is considered as the largest human gene located on chromosome Xp21 (Koenig 1987, Zhang 2013). Mutations can be inherited from female carriers who do not have any symptoms (~70%) or de novo (~30%) (Zhang 2013).

DMD is characterized by symptoms that occur at the age of 2 to 3 years with progressive muscle weakness (Sbiti 2002). Muscle weakness is the consequence of degeneration and necrosis of the proximal muscles, and calf pseudo-hypertrophy is shown in most patients with DMD at 2 or 3 years of age but can also be seen in infants. About 50% of boys with DMD start walking after 18 months (Gardner-Medwin 1978). In general, muscle weakness and difficulty in ambulation are first noted at the age between 2 and 3 years. This can be expressed as toe walking, difficulty running, climbing stairs, and frequently falling. Muscle weakness is expressed more in proximal

than in distal muscle groups and in the lower extremities more than in the upper extremities (Venugopal 2019).

Other symptoms associated with DMD include intellectual impairment (can be expressed with IQ less than 70 in 20% to 30% of cases, learning difficulties, autism-like behavior, and epilepsy), DMD-associated cardiomyopathy, lumbar lordosis and scoliosis, enlargement of the calves, and Trendelenburg gait (Venugopal 2019). Muscle weakness can lead to the loss of independent ambulation by the age of 12 years and death because of dilated cardiomyopathy (Bushby 2010a). Individuals with DMD usually die before the age of 25 years. The main leading causes of death are respiratory infections and insufficiency and cardiac failure (Mukoyama 1987, Patterson 1991).

Different types of genetic mutations are responsible for DMD. Large deletions spanning one or more exons are the most commonly identified; approximately 50% to 60% of individuals with DMD have this mutation (Prior 2005, Todorova 2008, Guo 2015, Yiu 2015). Another portion of individuals have point mutations (10%) (mostly nonsense), frameshift mutations (10% to 30%), duplications (5% to 15%), and intronic or 5′- and 3′-UTR alterations (2%) (Prior 2005, Nosaeid 2009, Zhang 2013, Wang 2014, Guo 2015, Yiu 2015). Commonly used genetic tests to identify mutations include multiplex polymerase chain reaction (PCR), multiplex ligation-dependent probe amplification (MLPA), Sanger sequencing test, and "next-generation" sequencing (NGS). Multiplex PCR is the least expensive but detects only deletions and does not cover the whole gene. MLPA will detect deletions and duplications and cover all exons, and sequencing tests (Sanger test and NGS) provide sequence data that help to determine smaller mutations, such as frameshift and nonsense mutations. The NGS technology enables the generation of large volumes of sequence more efficiently and cost effectively than traditional Sanger sequencing and is a promising screening tool (Bushby 2010a, Wang 2014).

Different aspects of DMD have been investigated in Saudi patients; however, the genetic characteristics, specifically mutations in the dystrophin gene, have not been thoroughly investigated. Research on the sphere of genetic characteristics, specifically on mutations in the dystrophin gene (Al Jumah 2002), patterns of dystrophin gene deletion/duplication (Friis 2014), the spectrum of deletions in the "hot-spot" regions of the DMD gene (Chaudhary 2008), as well as deletion mutations in children (Tayeb 2010), serves as a good starting point for further investigations into the genetic aspects of the Saudi DMD population (Mohammed 2018).

7.2. Study Rationale

A report of current management of DMD in the Middle East has identified the need for a multidisciplinary approach incorporating specific guidelines for diagnosis and treatment in the Middle East and North Africa (MENA) region to improve outcomes, increase disease awareness among the general public and the medical community, and establish general education programs regarding early signs and symptoms, a standardized referral and diagnosis pathway, patient registries, and support groups (Al Jumah 2019).

The current study aimed to investigate the genetic mutations in Saudi patients with DMD in the KSA based on the exploration of their demographic and clinical characteristics of the disease to further understand the relationship between genetic mutations, demographic data, and clinical manifestation.

8. STUDY OBJECTIVES

The primary objective of this study was to further the understanding of the relationship among genetic mutations, demographic data, and clinical manifestation in Saudi patients with DMD in KSA. The study objectives and endpoints are presented in Table 4. Details are provided in Section 9.5.

Table 4: Study Objectives and Endpoints

**Primary Objective**	**Primary Endpoint**
To describe the genetic mutations of 1- to 14-year-old male Saudi patients with a confirmed diagnosis of DMD disease in the KSA.	Genetic mutations in the DMD gene based on genetic test results from medical records
**Secondary Objectives**	**Secondary Endpoints**
1. To describe the demographics of 1- to 14-year-old male Saudi patients with a confirmed diagnosis of DMD disease in the KSA.<br>2. To describe the clinical characteristics/patient's phenotype of 1- to 14-year-old male Saudi patients with a confirmed diagnosis of DMD disease in the KSA.<br>3. To record the initial management plan for 1- to 14-year-old male Saudi patients with a confirmed diagnosis of DMD disease in the KSA.	Based on medical records, which include the following:<br>1. Demographics<br>2. Clinical characteristics/profile, including diagnosis, functional ability and status, medical history, and medications<br>3. Initial management plan details, including prescribed medications, physical therapy, and other methods

Abbreviations: DMD, Duchenne muscular dystrophy; KSA, Kingdom of Saudi Arabia

9. INVESTIGATIONAL PLAN

9.1. Overall Study Design and Plan: Description

This was an ambispective observational multicenter study conducted in collaboration with 9 study sites (hospitals) across the KSA that performed DMD genetic testing as per their routine clinical practice.

The study consisted of retrospective and prospective observations. The retrospective part of the study involved a review of medical records to identify all cases of DMD diagnosed between January 2010 and the date of the first site study initiation (18 August 2020). The prospective part of the study included observation of detected cases of DMD during the 18 months after the first site study initiation (18 August 2020) in the participant hospitals (Figure 1).

Figure 1: Study Design

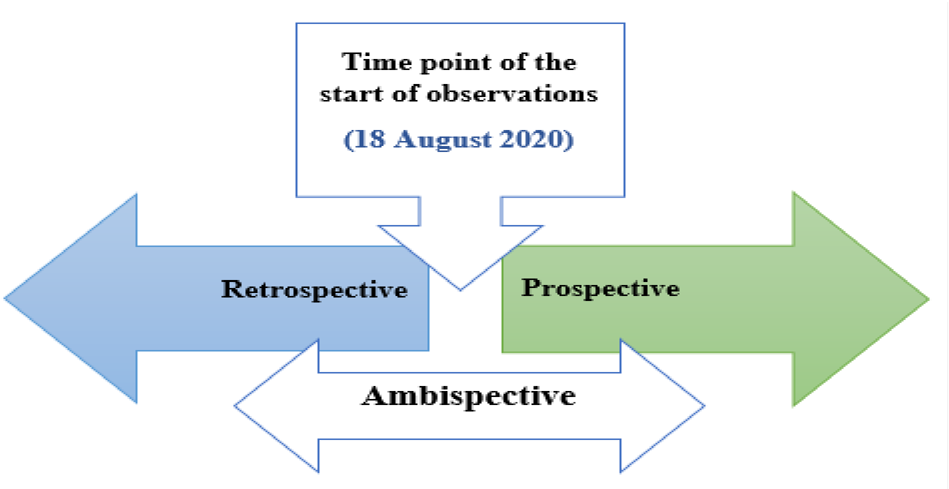

Data were collected at 1 timepoint for each patient in this study for both retrospective and prospective parts. No patients were evaluated both retrospectively and prospectively.

9.1.1. Retrospective Part

De-identified patient data were included in the retrospective part of the study. For de-identification, screening number (or ID) was assigned for each patient instead of initials. A list of screening numbers with corresponding initials was kept separately and used for patient identification if needed. The medical records of patients with genetically confirmed DMD were reviewed, and relevant information regarding genetic mutations, demographics, clinical characteristics, and the initial management plan were transferred to the electronic Case Report Form (eCRF) for further analyses.

At this visit, the physician-investigator completed the following information (evaluations that were conducted as per local usual clinical care and collected from medical charts) as study procedure:

1. Inclusion/exclusion criteria
2. Demographic variables, including height, where available
3. Medical history
4. DMD dystrophin mutation sequence data
5. Genetic mutations based on genetic test results, if available
6. Clinical characteristics/patient’s phenotype
7. Laboratory and histology test results
8. Initial management plan

9.1.2. Prospective Part

During the prospective part, after being aware of a diagnosed, genetically confirmed case of DMD, a site visit was initiated, and the Informed Consent Form was provided to the patient’s legal representative. For patients aged 12 to 14 years, an Informed Assent Form (IAF) was also

provided. Genetic testing results and medical records of diagnosed DMD patients were reviewed after consent was obtained, and relevant data were transferred to the eCRF as described above for the retrospective part (Section 9.1.1). The prospective part continued for 18 months.

9.2. Discussion of Study Design, Including the Choice of Control Groups

The chosen study design is an ambispective cohort study: a combination of prospective and retrospective cohort study that focuses on the past, as a baseline, with present and future to determine the occurrence of a disease and its evolution over time (Friis 2014). The study was designed to collect both retrospective and prospective data on genetic mutations, as well as demographics, clinical characteristics, and the initial management plan of male Saudi patients aged 1 to 14 years with a confirmed diagnosis of DMD disease, which is the sites' routine clinical practice.

9.3. Selection of Study Population

For retrospective patients during the screening process, eligibility criteria were checked based on medical record data, including genetic test results. Patients who did not satisfy all inclusion criteria and met any exclusion criteria were considered screen failures. For prospective patients, eligibility criteria also included informed consent, signed by the patient's legal representative, and IAF, signed by patients aged 12 to 14 years.

9.3.1. Inclusion Criteria

For inclusion into the study, patients were required to fulfill all of the following criteria:

1. Patient is a Saudi citizen.
2. Patient is a male between 1 to 14 years of age.
3. Patients whose legal guardians gave their consent for the prospective patients, while the retrospective patients were exempted from the requirement of consent.
4. Patients were able to give assent in the 12- to 14-year age range for the prospective patients, while the retrospective patients were exempted from the requirement of assent.
5. Patients had a genetically confirmed diagnosis record of DMD disease from January 2010 to 18 August 2020 for retrospective patients and 19 August 2020 to current for prospective patients.

9.3.2. Exclusion Criteria

Any of the following was regarded as a criterion for exclusion from the study:

1. Patients whose legal guardians were not able to provide informed consent (for prospective patients).
2. Patients 12 to 14 years of age who did not sign the assent form (for prospective patients).

9.3.3. Patient Withdrawal

Patients could be withdrawn from the study if, during data processing, it was found that they did not meet eligibility criteria (missed during the screening), or the patient's legal representative or patient withdrew consent/assent.

9.4. Treatments

No treatment was administered in this observational study.

9.5. Efficacy Variables and Schedule of Assessment

9.5.1. Schedule of Assessments

A schedule of the study procedures for both the retrospective and prospective parts of the study is provided in Table 5.

Table 5: Visit Schedule

Study Procedure	Retrospective Phase	Prospective Phase
**Timelines**	**At the Date of Diagnosis**	**At Enrollment**
Consent/assent (prospective patients)		x
Inclusion/exclusion	x	x
Demographic variables	x	x
Medical history	x	x
Genetic mutations (deletion, duplication, or point mutation)	x	x
Clinical characteristics/phenotype	x	x
Laboratory test results (serum CK level, ALT/AST level)	X	x
Initial management plan	x	x

Abbreviations: ALT, alanine aminotransferase; AST, aspartate aminotransferase; CK, creatine kinase

9.5.2. Primary Endpoint

The primary endpoint was the distribution of genetic mutations in the DMD gene of Saudi patients with a confirmed diagnosis of DMD disease. This assessment was based on results of genetic tests performed as routine clinical practice for DMD diagnosis recorded on medical records.

The following types of genetic mutations present in the dystrophin gene were collected:

- Large deletions
- Large duplications
- Small mutations
  - Nonsense mutations
  - Missense mutations
  - Splicing mutations
  - Deletion/insertion mutations
  - Other (if none of the above 4 subtypes or if unknown)

9.5.3. Secondary Endpoints

The following secondary endpoints were assessed based on medical records:

1. Demographics

- Age
- Height
- Region origin - current region

2. Clinical characteristics/profile (variables related to the clinical characteristics) for DMD patients with a confirmed diagnosis, including:
   - Diagnosis
     - Site of diagnosis (current treatment center - referring treatment center)
     - Calculated age at diagnosis
     - Age at first signs/symptoms
     - List of presenting symptoms
     - Biopsy date (if applicable)
     - Date of the confirming genetic test
   - Referral (Yes/No)
     - If Yes: age at referral, date of referral, and date first seen at the current treatment center
   - Walking status (if available) (Yes/No)
     - If Yes: (not walking by 15 months - delay on walking - difficulty on walking - walks on their toes - waddles - other)
   - Ambulation status: fully wheelchair bound (Yes/No)
     - If Yes: start date
   - Ability to run (Yes/No)
   - Ability to climb stairs (Yes/No)
   - Presence or absence of intellectual impairment, including:
     - Low IQ (less than 70)
     - Speech delay
     - Learning difficulties
     - Autism-like behavior
   - Presence or absence of DMD-associated cardiomyopathy
   - Presence or absence of lumbar lordosis and scoliosis
   - Presence or absence of enlargement of the calves
   - Presence or absence of Trendelenburg gait
   - Presence or absence of sleep apnea

- Serum creatine kinase (CK) level
- Alanine aminotransferase (ALT)/aspartate aminotransferase (AST) level
- Pulmonary function tests (if available) (Yes/No)

  If Yes:
  - Name and date of the most recent assessment
  - Result of the assessment: forced expiratory volume in one second (FEV1) - FVC - the ratio of the 2 volumes (FEV1/FVC)

3. Initial management plan
   - Medication data: name - dose - frequency - route - start date - end date
     - Corticosteroids
     - Calcium and vitamin D use as prophylaxis
     - Other
   - Physical therapy
   - Surgery
   - Device
   - Diet
   - Educational and psychological intervention

9.6. Data Quality Assurance

In accordance with local regulations and relevant International Conference on Harmonization guidelines, PTC Therapeutics (PTC) or a designee periodically inspected all eCRFs and study documents associated with this study at mutually convenient times, before, during, and after completion of the study. These reviews allowed for verification of the accuracy and completeness of data in the eCRFs and assurance that all protocol requirements, relevant regulations, and investigator’s obligations were being fulfilled.

9.7. Statistical Methods Planned in the Protocol and Determination of Sample Size

9.7.1. Statistical and Analytical Plans

As this was a descriptive study, summary statistics, including the mean, standard deviation, median, range, and interquartile range, were used for numeric variables such as age, and frequency distributions were used for categorical variables such as gender and type of mutation. Missing data for categorical variables were counted and presented as a separate category if it constituted at least 10% of the sample size obtained. For numeric variables, the total number of valid answers was presented next to the variable name in the created tables. IBM-SPSS was used for all analyses.

9.7.1.1. *Baseline Descriptive Statistics*

All descriptive statistics were considered baseline in this cross-sectional study as no follow-up assessments were planned. The variables were analyzed as described in Section 9.7.1.

9.7.1.2. *Analysis of Primary and Secondary Endpoints*

For the primary efficacy endpoints, the prevalence of each type of genetic mutation was computed along with its Wald 95% confidence intervals or Clopper Pearson's confidence interval when needed.

Secondary endpoints such as age at diagnosis and presence or absence of muscle weakness were summarized depending on the type of the variable as described in Section 9.7.1.

9.7.1.3. *Safety Analyses*

There was no safety analysis planned for this study since it was descriptive in nature, and no treatment was administered.

9.7.1.4. *Planned Interim Analyses*

An interim analysis was conducted to evaluate the demographics and clinical characteristics of patients included retrospectively between January 2014 and September 2020 and prospectively between September 2020 and April 2021. At the time of the interim analysis (data cutoff 20 April 2021), 177 of 188 enrolled patients were included in the analysis (AlSaman 2022).

9.7.2. Determination of Sample Size

The sample size was based on the estimation of the different types of mutations, the primary outcome among the studied population. Thus, this would be based on establishing 95% confidence intervals for the prevalence of the different types of mutations. The confidence interval for a prevalence has the formula p±MOE, where MOE is the margin of error. Table 6 displays the maximum MOEs associated with those confidence intervals based on different sample sizes and the MOEs based on a prevalence of 25% and 15%.

Table 6: Sample Size Estimations Based on Mutation Prevalence

Sample Size	100	150	200	250
Maximum margin of error (±)	10%	8%	7%	6.3%
Margin of error with 25% prevalence	8.5%	7%	6%	5.5%
Margin of error with 15% prevalence	7.3%	5.8%	5%	4.5%

The study aimed to capture retrospectively and prospectively every genetically confirmed DMD patient in all 9 study sites from January 2010 to 18 months after the start of the study. It was estimated based on input from the study sites that between 150 and 210 patients would be identified, and thus, the MOEs with the prevalence were similar to those indicated above.

9.8. Changes in the Conduct of the Study or Planned Analyses

The original protocol was amended 4 times during the study. Versions 1 and 2 were not implemented at any site. The majority of the substantial protocol changes were implemented in protocol versions 4 and 5. A summary of the key changes in these versions is presented in Table 7.

Table 7: Summary of Study PTC-GD-MA-405 Substantive Protocol Changes

Version (Date)	Brief Summary of Substantive Changes
Version 5.0 (30 September 2021)	Added details of mutations to be collected
	Increased sample size up to 270 patients
	Clarified time period for genetically confirmed diagnosis record of DMD disease from January 2010 to 18 August 2020 for retrospective patients and 19 August 2020 and onward for prospective patients
	Added collection of height to demographic variables<br>Added collection of predicted FVC to clinical laboratory and histology results<br>Added collection of DMD dystrophin mutation sequence data
	Allowed for verbal consent during the prospective period to accommodate COVID-19 restrictions
Version 4.0 (18 March 2021)	Removed race and ethnicity collection
	Added collection of region origin/current region within the demographics data
	Added collection of clinical characteristics and initial management plan data
	Modified retrospective part to identify all cases of DMD diagnosed between January 2010 and the date of the first site study initiation (18 August 2020)
	Increased the prospective recruitment from 12 months to 18 months
	Added option for interim analysis
	Increased the estimated sample size to 210 patients

Abbreviations: COVID-19, coronavirus disease 2019; DMD, Duchenne muscular dystrophy; FVC, forced vital capacity

10. STUDY PATIENTS

10.1. Disposition of Patients

Of the 253 patients identified, 226 patients met eligibility criteria and were included in the study (Table 8). A total of 181 patients were enrolled in the retrospective part; 45 patients were enrolled in the prospective part.

Most (20) of the 27 identified patients who were excluded from the study were not eligible because their date of genetic confirmation of DMD was outside of the inclusion range. Six patients were outside of the age range of 1 to 14 years; 1 patient was not a citizen of Saudi Arabia (Table 14.2).

Table 8: Patient Disposition/Patient Completion Summary

	N	%
Enrolled	253	100.0%
Eligible	226	89.3%
Excluded	27	10.7%

Source: Table 14.1

10.2. Demographics and Other Baseline Characteristics

Most of the patients were from the Makkah (20%) and Riyadh (18.7%) regions (Table 9).

Table 9: Summary of Demographics

Variable	All Patients
**Region of Origin, n (%)**	**N=225**
Makkah	45 (20.0)
Riyadh	42 (18.7)
Asser	20 (8.9)
Eastern Region	20 (8.9)
Madinah	17 (7.6)
Jazan	16 (7.1)
Northern Borders	14 (6.2)
Al Baha	13 (5.8)
Najran	12 (5.3)
Tabuk	10 (4.4)
Ha'il	7 (3.1)
Qassim	7 (3.1)
Al Jawf	2 (0.9)
**Current Region, n (%)**	**N=218**
Makkah	58 (26.6)
Riyadh	48 (22.0)
Eastern Region	30 (13.8)
Asser	17 (7.8)
Madinah	12 (5.5)
Northern Borders	12 (5.5)
Najran	10 (4.6)
Jazan	8 (3.7)
Tabuk	7 (3.2)
Qassim	6 (2.8)
Ha'il	5 (2.3)
Al Baha	4 (1.8)
Al Jawf	1 (0.5)

Source: Table 14.3

The most common genetic mutation among the 226 enrolled patients was a large deletion (134 patients, 59.3%). Approximately 30% of patients had small mutations, including 39 patients (17.3%) with a nonsense mutation (Table 10). These data are generally consistent with the reported global distribution of DMD mutations (Bladen 2015).

Table 10: Genetic Mutations

Mutation	Retrospective (n=181)		Prospective (n=45)		Overall (n=226)	
	N (%)	95% Confidence Interval	N (%)	95% Confidence Interval	N (%)	95% Confidence Interval
Large deletion	104 (57.5)	49.9, 64.8	30 (66.7)	51.0, 80.0	134 (59.3)	52.6, 65.8
Large duplication	23 (12.7)	8.2, 18.5	2 (4.4)	0.5, 15.1	25 (11.1)	7.3, 15.9
Small mutations[a]	54 (29.8)	23.3, 37.1	13 (28.9)	16.4, 44.3	67 (29.6)	23.8, 36.1
Nonsense mutations	35 (19.3)	13.9, 25.9	4 (8.9)	2.5, 21.2	39 (17.3)	12.6, 22.8
Deletion/ insertion mutations	16 (8.8)	5.1, 14.0	9 (20.0)	9.6, 34.6	25 (11.1)	7.3, 15.9
Splicing mutations	3 (1.7)	0.3, 4.8	0	0.0, 7.9	3 (1.3)	0.3, 3.8
Other mutations	3 (1.7)	0.3, 4.8	0	0.0, 7.9	3 (1.3)	0.3, 3.8
2 or more small mutations	3 (1.7)	0.3, 4.8	0	0.0, 7.9	3 (1.3)	0.3, 3.8

[a] Three patients had 2 small mutations.
Source: Table 14.4

Although 80% of patients were younger than 5 years at the time of first symptoms, fewer than 30% of patients had received a DMD diagnosis before the age of 5 years. The majority of patients (61.5%) were referred from other centers. Approximately one third of patients were not walking by the age of 18 months. (Table 11).

Table 11: Duchenne Muscular Dystrophy Characteristics

Variable	Retrospective Patients (N=181)	Prospective Patients (N=45)	Total (N=226)
**Age at diagnosis (years), n (%)**	**N=181**	**N=45**	**N=226**
<2	2 (1.1)	0	2 (0.9)
≥2 to <5	44 (24.3)	13 (28.9)	57 (25.2)
≥5 to 14	135 (74.6)	32 (71.1)	167 (73.9)
**Age at diagnosis (years)**	**N=181**	**N=45**	**N=226**
Mean (SD)	7.0 (2.8)	6.7 (2.5)	6.9 (2.7)
Median (Q1, Q3)	6.9 (4.9, 8.6)	7.2 (4.5, 8.2)	7.0 (4.8, 8.5)
Min, Max	1.3, 13.8	2.3, 12.9	1.3, 13.8
**Age at first sign and symptoms (years), n (%)**	**N=97**	**N=45**	**N=142**
<2	21 (21.6)	14 (31.1)	35 (24.6)
≥2 to <5	54 (55.7)	25 (55.6)	79 (55.6)
≥5 to 14	22 (22.7)	6 (13.3)	28 (19.7)
**Age at first sign and symptoms (years)**	**N=97**	**N=45**	**N=142**
Mean (SD)	3.8 (2.3)	3.2 (2.3)	3.6 (2.3)
Median (Q1, Q3)	2.9 (2.0, 4.9)	2.5 (1.9, 3.5)	2.7 (2.0, 4.6)
Min, Max	0.4, 10.3	0.6, 10.6	0.4, 10.6
**Age at walking, n (%)**	**N=150**	**N=45**	**N=195**
Not walking by 15 months	56 (37.3)	19 (42.2)	75 (38.5)
Not walking by 18 months	46 (30.7)	11 (24.4)	57 (29.2)
**Site of diagnosis, n (%)**			
Current treatment center	143 (79.0)	32 (71.1)	175 (77.4)
Referring treatment center	31 (17.1)	13 (28.9)	44 (19.5)
Not available	7 (3.9)	0	7 (3.1)
**Referral from another center, n (%)**			
Yes	111 (61.3)	28 (62.2)	139 (61.5)
No	47 (26.0)	17 (37.8)	64 (28.3)
Not available	23 (12.7)	0	23 (10.2)

Abbreviations: Max, maximum; Min, minimum; SD, standard deviation
Source: Table 14.5

Consistent with the trend observed in the interim analysis, the rates of symptom identification (Figure 2) and DMD diagnosis (Figure 3) increased over time and generally peaked between 2014 and 2018, reflecting increased awareness of DMD on the part of healthcare providers and parents and availability of genetic testing. Declines seen after 2019 are likely caused by plateauing of the data and the effect of COVID-19 restrictions on hospital visits.

Figure 2: Year of First Signs and Symptoms of DMD

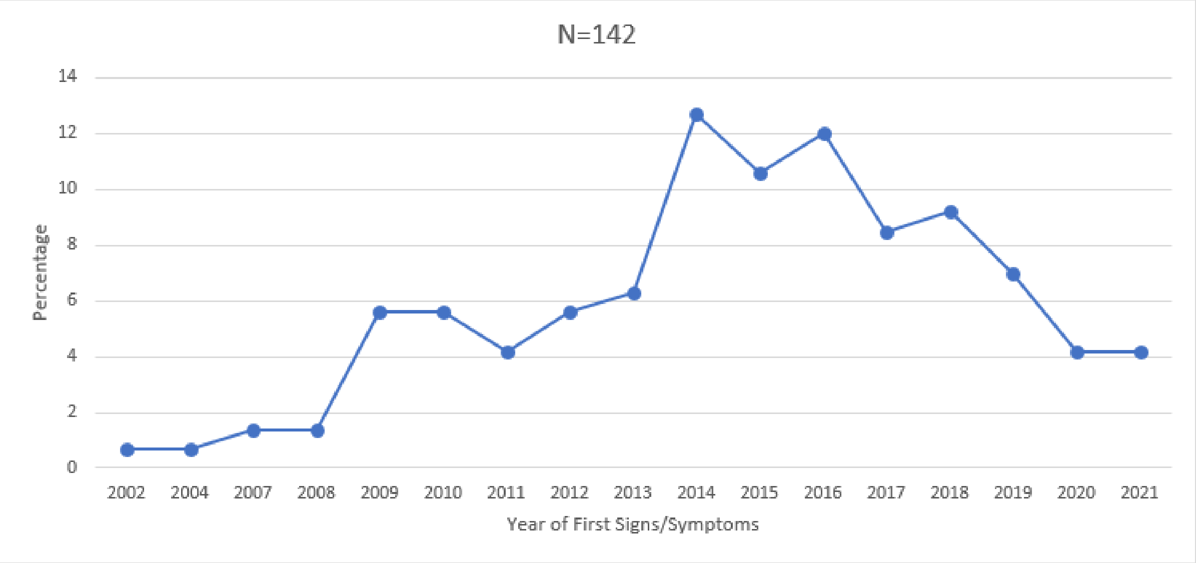

Abbreviations: DMD, Duchenne muscular dystrophy
Source: Table 14.5

Figure 3: Year of Diagnosis of DMD

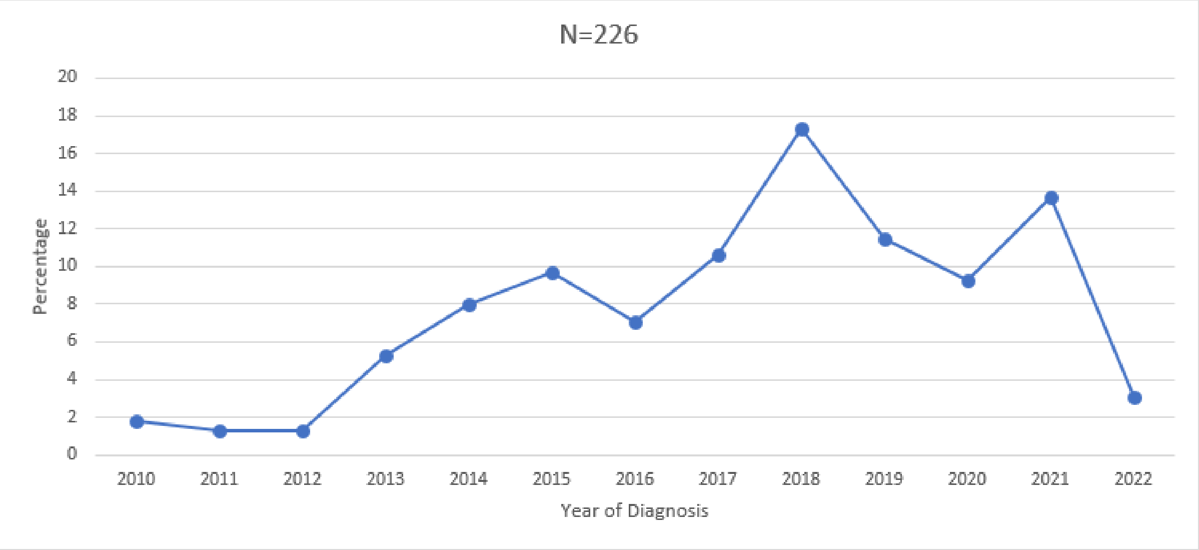

Abbreviations: DMD, Duchenne muscular dystrophy
Source: Table 14.5

The great majority of patients (195 [86.3%]) had walking characteristics of DMD (Table 12). Among these patients, the most common observed characteristic was difficulty on walking (87.7%) and waddling gait (41%).

Across all 226 patients, 64 (28.3%) were fully wheelchair bound at the time of study entry, primarily in the retrospective part of the study. This is consistent with the trend toward earlier diagnosis and increased genetic testing. The mean age at loss of ambulation for patients who

were fully wheelchair bound was 10.4 years. Among the ambulatory patients, 55 (43.3%) were able to run and 75 (59.1%) were able to climb.

Table 12: Walking Characteristics

Variable	Retrospective Patients (N=181)	Prospective Patients (N=45)	Total (N=226)
**Present walking characteristics, n (%)**			
Yes	150 (82.9)	45 (100)	195 (86.3)
No	5 (2.8)	0	5 (2.2)
Not available	26 (14.4)	0	26 (11.5)
**Walking characteristics[a], n (%)**			
Difficulty on walking	131 (87.3)	40 (88.9)	171 (87.7)
Walks on their toes	28 (18.7)	16 (35.6)	44 (22.6)
Waddles	54 (36.0)	26 (57.8)	80 (41.0)
Others	9 (6.0)	2 (4.4)	11 (5.6)
**Patient fully wheelchair bound, n (%)**			
Yes	60 (33.1)	4 (8.9)	64 (28.3)
No	86 (47.5)	41 (91.1)	127 (56.2)
Not available	35 (19.3)	0	35 (15.5)
Missing	0	0	0
**Age at fully wheelchair bound (years)**	**N=27**	**N=3**	**N=30**
Mean (SD)	10.5 (2.4)	9.7 (1.6)	10.4 (2.3)
Median (Q1, Q3)	10.7 (9.3, 12.1)	9.0 (8.6, 11.6)	10.6 (9.1, 11.6)
Min, Max	5.0, 15.5	8.6, 11.6	5.0, 15.5
**Patient able to run,[b] n (%)**			
Yes	38 (44.2)	17 (41.5)	55 (43.3)
No	30 (34.9)	24 (58.5)	54 (42.5)
Not available	18 (20.9)	0	18 (14.2)
Missing	0	0	0
**Patient able to climb stairs,[b] n (%)**			
Yes	51 (59.3)	24 (58.5)	75 (59.1)
No	26 (30.2)	17 (41.5)	43 (33.9)
Not available	9 (10.5)	0	9 (7.1)
Missing	0	0	0

Abbreviations: Max, maximum; Min, minimum; SD, standard deviation

[a] Percentages computed among those who had present walking characteristics.

[b] Percentages were computed among those who were not fully wheelchair bound.

Source: Table 14.5

10.3. Clinical Characteristics

The most commonly reported clinical characteristics were calf enlargement (86.7%), lumbar lordosis (40.9%), and lumbar scoliosis (32.5%). Pulmonary function tests data were unavailable for most patients in the retrospective part, but the data were suggestive of greater pulmonary function among the patients in the prospective part. Among the patients with available laboratory data, serum CK, ALT, and AST were elevated, which is consistent with DMD disease (Table 13).

Table 13: Clinical Characteristics

Variable	Retrospective Patients (N=181)	Prospective Patients (N=45)	Total (N=226)
**Clinical characteristics[a], n (%)**	**N=163**	**N=40**	**N=203**
Enlargement of the calves	137 (84.0)	39 (97.5)	176 (86.7)
Lumbar lordosis	65 (39.9)	18 (45.0)	83 (40.9)
Lumbar scoliosis	51 (31.3)	15 (37.5)	66 (32.5)
Trendelenburg gait	40 (24.5)	19 (47.5)	59 (29.1)
Sleep apnea	1 (0.6)	0	1 (0.5)
DMD-associated cardiomyopathy	9 (5.5)	0	9 (4.4)
Other	7 (4.3)	2 (5.0)	9 (4.4)
**Intellectual impairment, n (%)**			
Yes	46 (25.4)	11 (24.4)	57 (25.2)
No	97 (53.6)	33 (73.3)	130 (57.5)
Not available	38 (21.0)	1 (2.2)	39 (17.3)
Missing	0	0	0
**Pulmonary function tests, n (%)**			
Yes	19 (10.5)	2 (4.4)	21 (9.3)
No	97 (53.6)	43 (95.6)	140 (61.9)
Not available	65 (35.9)	0	65 (28.8)
**FEV1 (L)**			
Mean (SD)	1.0 (0.0)	1.5 (0.7)	1.0 (0.2)
Median	1.0	1.5	1.0
Min, Max	1.0, 1.0	1.0, 2.0	1.0, 2.0
**FVC (L)**			
Mean (SD)	1.1 (0.2)	1.5 (0.7)	1.1 (0.3)
Median	1.0	1.5	1.0
Min, Max	1.0, 2.0	1.0, 2.0	1.0, 2.0
**FEV1/FVC (%)**			
Mean (SD)	84.1 (16.7)	98.5 (20.5)	85.5 (17.1)
Median	78	98.5	79.0
Min, Max	68.0, 116.0	84.0, 113.0	68.0, 116.0
**Predicted FVC (L)**			
Mean (SD)	4.5 (14.7)	36.0 (49.5)	7.5 (20.1)
Median	1.0	36	1.0
Min, Max	1.0, 65.0	1.0, 71.0	1.0, 71.0

Variable	Retrospective Patients (N=181)	Prospective Patients (N=45)	Total (N=226)
**ALT (U/L)**	**N=115**	**N=21**	**N=136**
Mean (SD)	348.6 (186.6)	334.1 (165.4)	346.4 (182.9)
Median	332.0	327.0	331.0
Min, Max	36.0, 1181.0	81.0, 746.0	36.0, 1181.0
**AST (U/L)**	**N=99**	**N=18**	**N=117**
Mean (SD)	246.7 (154.3)	244.1 (158.5)	246.3 (154.3)
Median	211.0	189.5	210.0
Min, Max	48.0, 967.0	45.0, 623.0	45.0, 967.0
**CK (U/L)**	**N=147**	**N=27**	**N=174**
Mean (SD)	12585.3 (7625.4)	12336.6 (7795.4)	12546.7 (7629.8)
Median	11017.0	11350.0	11051.0
Min, Max	759.0, 50953.0	1100.0, 29535.0	759.0, 50953.0

Abbreviations: ALT, alanine aminotransferase; AST, aspartate aminotransferase; CK, creatine kinase; DMD, Duchenne muscular dystrophy; FEV1, forced expiratory volume in one second; FVC, forced vital capacity; Max, maximum; Min, minimum; SD, standard deviation

[a] Percentages did not add up to 100, as a patient might have multiple present clinical characteristics.

Source: Table 14.5

10.4. Initial Management Plan

The initial management plans for most patients primarily involved medication and physical therapy (Figure 4 and Table 14.5).

Figure 4: Components of Initial Management Plan

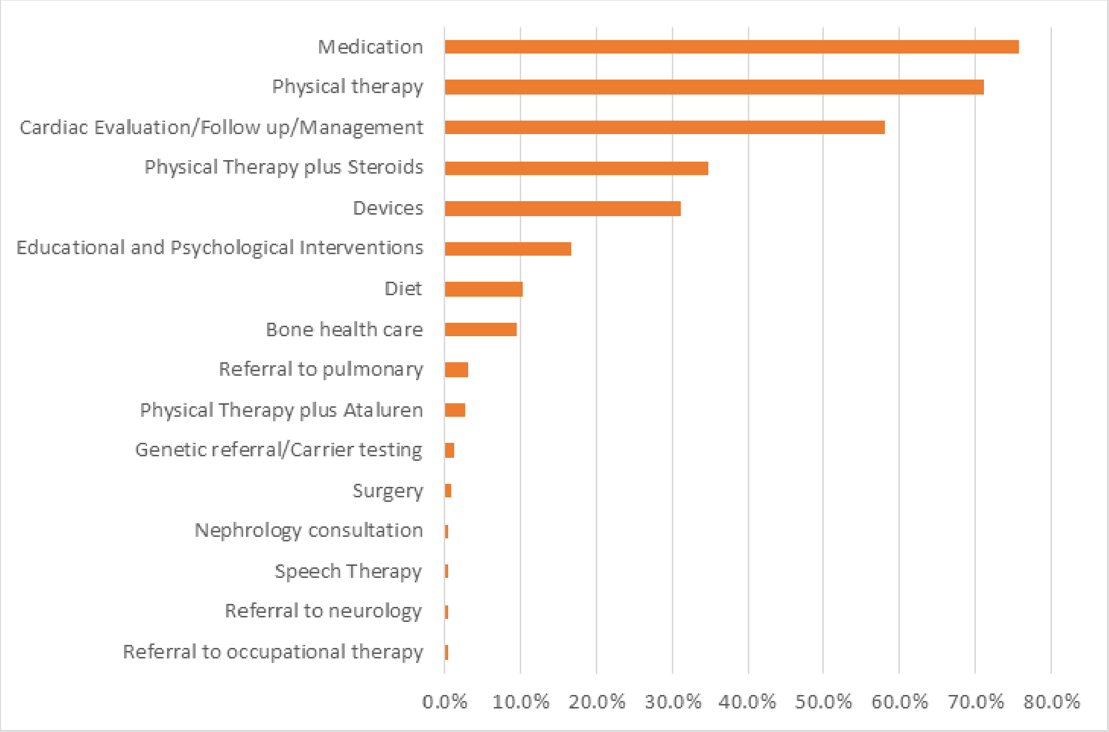

Source: Table 14.5

The most frequently prescribed initial medications were vitamin D (82%) and steroids (62.3%). Ataluren, which is indicated for the treatment of nonsense mutation Duchenne muscular dystrophy (nmDMD), was included in the initial management plan for only 6 patients. Although few patients in the prospective part of the study have data regarding the age at initiation of steroid use, the results suggest a trend toward initiation of corticosteroid treatment at an earlier age (Table 14). This is consistent with the current standard of care for DMD that recognizes the benefits of corticosteroid treatment (Moxley 2010).

Table 14: Initial Management Plan

Variable	Retrospective Patients (N=181)	Prospective Patients (N=45)	Total (N=226)
**Age at steroid initiation (years)**	**N=78**	**N=11**	**N=89**
Mean (SD)	7.5 (2.4)	6.7 (1.9)	7.4 (2.3)
Median (Q1, Q3)	7.3 (5.9, 8.7)	6.6 (4.8, 8.2)	7.1 (5.7, 8.7)
Min, Max	2.6, 14.2	4.2, 10.2	2.6, 14.2
**Age group (years) at steroid initiation, n (%)**			
≥2 to <5	13 (16.7)	3 (27.3)	16 (18.0)
≥5 to <7	22 (28.2)	4 (36.4)	26 (29.2)
≥7	43 (55.1)	4 (36.4)	47 (52.8)
**Age at ataluren initiation (years)**	**N=6**		**N=6**
Mean (SD)	7.2 (2.4)	-	7.2 (2.4)
Median (Q1, Q3)	7.5 (5.7, 8.8)	-	7.5 (5.7, 8.8)
Min, Max	2.9, 9.9	-	2.9, 9.9
**Age group (years) at ataluren initiation, n (%)**			
<7	2 (33.3)	-	2 (33.3)
≥7	4 (66.7)	-	4 (66.7)

Abbreviations: Max, maximum; Min, minimum; SD, standard deviation
Source: Table 14.5

As shown in Figure 5 and Table 15, the use of corticosteroids and physical therapy, both standards of care in many global regions, was inconsistent across the clinical sites.

Figure 5: Physical Therapy Management by Clinical Site and Steroid Treatment

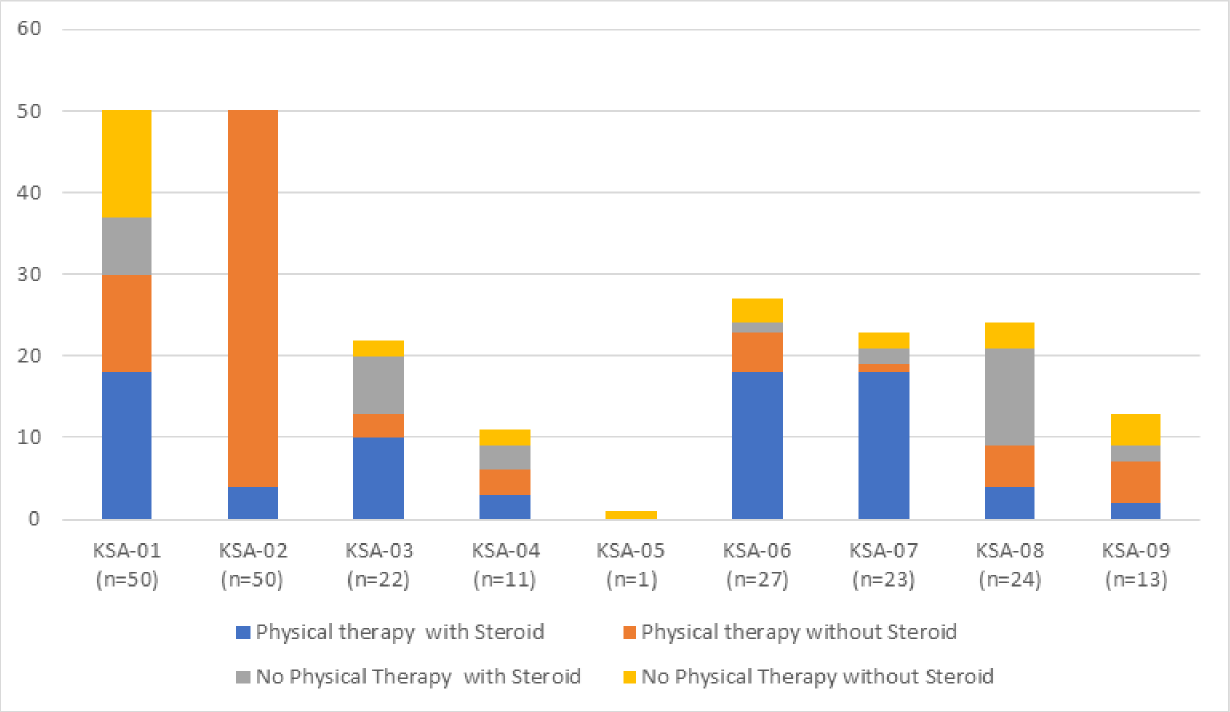

Source: Table 14.7

Across all 226 patients, 157 patients received physical therapy as part of the initial management plan, and 111 patients reported receiving corticosteroid treatment.

Table 15: Summary of Physical Therapy Management by Site and Steroid Treatment

Site	Physical Therapy = Yes		Physical Therapy = No	
	With Steroid (n=77)	Without Steroid (n=80)	With Steroid (n=34)	Without Steroid (n=30)
	n (%)			
KSA-01	18 (23.4)	12 (15.0)	7 (20.6)	13 (43.3)
KSA-02	4 (5.2)	46 (57.5)	0	0
KSA-03	10 (13.0)	3 (3.8)	7 (20.6)	2 (6.7)
KSA-04	3 (3.9)	3 (3.8)	3 (8.8)	2 (6.7)
KSA-05	0	0	0	1 (3.3)
KSA-06	18 (23.4)	5 (6.3)	1 (2.9)	3 (10.0)
KSA-07	18 (23.4)	1 (1.3)	2 (5.9)	2 (6.7)
KSA-08	4 (5.2)	5 (6.3)	12 (35.3)	3 (10.0)
KSA-09	2 (2.6)	5 (6.3)	2 (5.9)	4 (13.3)

Note: N=221 as 5 patients had no reported management plans.
Source: Table 14.7

All but 9 of the patients who received corticosteroids were receiving prednisolone. Seven patients (6.3%) were receiving prednisone, and 2 patients (1.8%) were receiving deflazacort (Table 14.5.1).

11. EFFICACY EVALUATION

This study evaluated the relationship between the age at diagnosis, initial management plan (standard of care), and age at initiation of treatment on disease outcomes in patients with DMD.

11.1. Effect of Medication on Physical Function

As shown in Table 16, patients who were prescribed corticosteroid treatment were less likely to be fully wheelchair bound (27% versus 37%). And although only 5 patients received corticosteroids with ataluren, the data suggest that the combination may be associated with better outcomes than the standard of care alone.

Table 16: Ambulation Characteristics of the Included Patients According to Types of Prescribed Active Medication

		Type of Treatment		
		**Steroid Only (n=104)**	**Steroid + Ataluren (n=5)**	**No Steroid or Ataluren (n=54)**
		**n (%)**		
**Fully wheelchair bound**	Yes	29 (27.9)	1 (20.0)	20 (37.0)
	No	71 (68.3)	4 (80.0)	29 (53.7)
	Not available	4 (3.8)	0	5 (9.3)
**Able to run***	Yes	30 (42.3)	2 (50.0)	13 (44.8)
	No	30 (42.3)	2 (50.0)	11 (37.9)
	Not available	11 (15.5)	0	5 (17.2)
**Able to climb stairs***	Yes	39 (54.9)	4 (100)	18 (62.1)
	No	27 (38.0)	0	8 (27.6)
	Not available	5 (7.0)	0	3 (10.3)

* Among those not fully in wheelchair.
Source: Table 14.6.1

Although only sparse data were available, the benefits of interventional treatment were also evident in the age at loss of ambulation. The mean age at the time of being fully wheelchair bound was 10.3 years among patients who received corticosteroids compared to 9.6 years for patients who did not receive interventional therapy (Table 17). This is consistent with the published literature that has identified a benefit of corticosteroid treatment in delaying the loss of ambulation (Moxley 2010, McDonald 2018).

Table 17: Age at Fully Wheelchair Bound by Type of Treatment

Among Those Who Are Wheelchair Bound		Type of Treatment		
		Steroid Only (n=29)	Steroid + Ataluren (n=1)	No Steroid or Ataluren (n=20)
Age at fully wheelchair bound	n	15	1	8
	Mean (SD)	10.3 (2.3)	10.8 (0)	9.6 (2.7)
	Median (Q1, Q3)	10.1 (9.3, 11.2)	10.8 (10.8, 10.8)	9.8 (8.0, 11.4)
	Min, Max	4.9, 15.5	10.8, 10.8	5.0, 13.6
	n missing	14	0	12

Abbreviations: Max, maximum; Min, minimum; SD, standard deviation
* Among those fully in wheelchair.
Source: Table 14.6.1.1

11.2. Effect of Age at Diagnosis and Intervention on Physical Function

The assessment of the age at start of treatment and diagnosis highlights the importance of early diagnosis and intervention to delay disease progression and preserve physical functioning in patients with DMD.

11.2.1. Age at Diagnosis

With a progressive uniformly fatal disease like DMD, early diagnosis and intervention have been shown to be critical to improving outcomes and delaying disease milestones (Birnkrant 2018). As shown in Table 18, patients whose DMD was diagnosed before the age of 5 years were much more likely to retain ambulation than those who were diagnosed after the age of 5 years.

Table 18: Ambulation Characteristics by Age at Diagnosis

	Category	Age at Diagnosis		
		<5 Years (n=59)	≥5 Years (n=167)	Total (n=226)
		n (%)		
**Fully wheelchair bound**	Yes	6 (10.2)	58 (34.7)	64 (28.3)
	No	45 (76.3)	82 (49.1)	127 (56.2)
	Not available	8 (13.6)	27 (16.2)	35 (15.5)
**Able to run***	Yes	23 (51.1)	32 (39.0)	55 (43.3)
	No	12 (26.7)	42 (51.2)	54 (42.5)
	Not available	10 (22.2)	8 (9.8)	18 (14.2)
**Able to climb stairs***	Yes	27 (60.0)	48 (58.5)	75 (59.1)
	No	13 (28.9)	30 (36.6)	43 (33.9)
	Not available	5 (11.1)	4 (4.9)	9 (7.1)

* Among those not fully in wheelchair.
Source: Table 14.6.3

11.2.2. Age at Start of Active Treatment

Among 90 patients for whom age at the start of active treatment was available, outcomes were significantly better for patients who initiated treatment before the age of 7 years (Table 19 and Figure 6). Approximately half of the 47 patients who initiated treatment after the age of 7 years (23 patients, 48.9%) were fully wheelchair bound compared to 4 patients (9.3%) who initiated treatment before the age of 7 years.

Table 19: Ambulation Characteristics of the Included Patients According to Age at the Start of Treatment

		Age at Start of Treatment		
		<7 Years (n=43)	≥7 Years (n=47)	Total (n=90)
		n (%)		
**Fully wheelchair bound**	Yes	4 (9.3)	23 (48.9)	27 (30.0)
	No	36 (83.7)	23 (48.9)	59 (65.6)
	Not available	3 (7.0)	1 (2.1)	4 (4.4)
**Able to run***	Yes	17 (47.2)	11 (47.8)	28 (47.5)
	No	10 (27.8)	10 (43.5)	20 (33.9)
	Not available	9 (25.0)	2 (8.7)	11 (18.6)
**Able to climb stairs***	Yes	24 (66.7)	16 (69.6)	40 (67.8)
	No	8 (22.2)	6 (26.1)	14 (23.7)
	Not available	4 (11.1)	1 (4.3)	5 (8.5)

* Among those not fully in wheelchair.
Source: Table 14.6.2

Figure 6: Bar Chart of Ambulation Characteristics by Age at the Start of Treatment

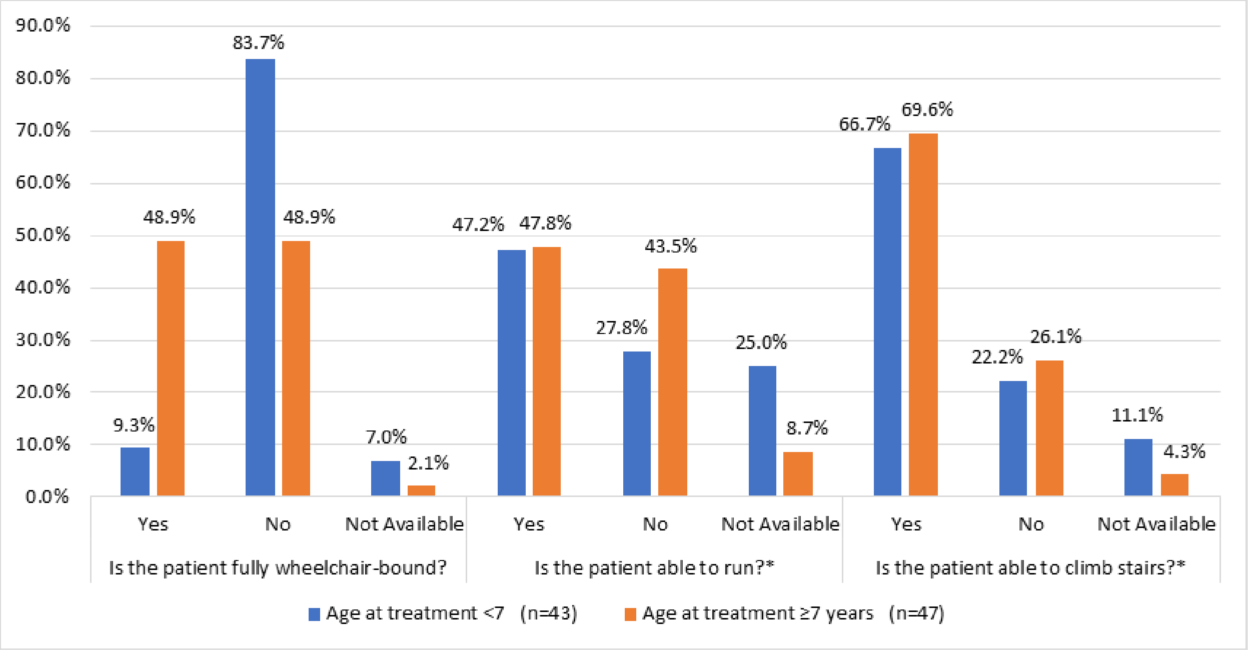

* Among those not fully in wheelchair.
Source: Table 14.6.2

12. SAFETY EVALUATION

Not applicable. This was a non-interventional study.

13. DISCUSSION AND OVERALL CONCLUSIONS

This ambispective cohort study evaluated genetic mutations, demographics, clinical characteristics, and initial management plans data for 226 male patients with DMD between the

ages of 1 and 14 years. Through collection of retrospective and prospective data, the study evaluated DMD standard of care and its evolution over time in Saudi Arabia. Information was collected from medical records available through the routine clinical practice at 9 centers in the KSA.

Consistent with the global prevalence of specific genetic mutations, most Saudi patients had a large deletion, representing approximately 60% of the cases. Approximately one third of patients had small mutations, half of which were nonsense mutations amendable to readthrough therapies.

While the majority of data were collected retrospectively, a comparison of retrospective and prospective data demonstrated a trend toward increasing rates of diagnosis over time, which likely reflects increasing awareness of the disease and the availability of genetic testing in Saudi Arabia. However, the data also indicate that DMD diagnosis in Saudi Arabia (median age of 7 years) still occurs later than the global average (Vry 2016). This in turn results in delays in the initiation of intervention, which has been shown to delay critical milestones and prolong survival (Birnkrant 2018). Indeed, in this study, initiation of treatment at a younger age was associated with prolongation of ambulation.

The effects of active medication on disease progression were evident in this study. Patients who were prescribed corticosteroid treatment were less likely to be fully wheelchair bound, which is consistent with the established benefit of corticosteroid treatment on preserving ambulation (Moxley 2010, McDonald 2018). And while only 5 patients received corticosteroids and ataluren, the data suggest that the combination of ataluren with standard of care may be associated with better outcomes than those from the standard of care alone.

This study has characterized the demographics and clinical profiles of patients with DMD in Saudi Arabia with the goal of increasing disease awareness and improving management of patients with DMD. While a trend toward increasing and earlier diagnosis and intervention were observed over time, regional inconsistencies highlight the continuing unmet need for education and standard management approaches to improve outcomes in patients with DMD in Saudi Arabia.

14. SUPPORTING TABLES

Table 14.1: Patient Disposition

Population	N	%
**Enrolled**	253	100.0%
**Eligible**	226	89.3%
**Excluded**	27	10.7%

Table 14.2: List of Excluded Patients and Causes of Exclusion

Patient ID	Cause of exclusion
KSA-01-017	Patient is not a Saudi citizen
KSA-01-054	Patient is not in the age range 1 to 14
KSA-06-013	Patient is not in the age range 1 to 14
KSA-06-020	Patient is not in the age range 1 to 14
KSA-07-020	Patient is not in the age range 1 to 14
KSA-07-021	Patient is not in the age range 1 to 14
KSA-08-029 / KSA-09-012	Patient is not in the age range 1 to 14
KSA-03-019	Date of genetically confirmed diagnosis of DMD is out of range for inclusion
KSA-04-009	Date of genetically confirmed diagnosis of DMD is out of range for inclusion
KSA-04-010	Date of genetically confirmed diagnosis of DMD is out of range for inclusion
KSA-06-003	Date of genetically confirmed diagnosis of DMD is out of range for inclusion
KSA-06-006	Date of genetically confirmed diagnosis of DMD is out of range for inclusion
KSA-06-010	Date of genetically confirmed diagnosis of DMD is out of range for inclusion
KSA-06-011	Date of genetically confirmed diagnosis of DMD is out of range for inclusion
KSA-08-006	Date of genetically confirmed diagnosis of DMD is out of range for inclusion
KSA-08-007	Date of genetically confirmed diagnosis of DMD is out of range for inclusion
KSA-08-009	Date of genetically confirmed diagnosis of DMD is out of range for inclusion
KSA-08-012	Date of genetically confirmed diagnosis of DMD is out of range for inclusion
KSA-08-020	Date of genetically confirmed diagnosis of DMD is out of range for inclusion
KSA-08-022	Date of genetically confirmed diagnosis of DMD is out of range for inclusion
KSA-08-023	Date of genetically confirmed diagnosis of DMD is out of range for inclusion
KSA-08-026	Date of genetically confirmed diagnosis of DMD is out of range for inclusion

Patient ID	Cause of exclusion
KSA-08-027	Date of genetically confirmed diagnosis of DMD is out of range for inclusion
KSA-08-030	Date of genetically confirmed diagnosis of DMD is out of range for inclusion
KSA-08-038	Date of genetically confirmed diagnosis of DMD is out of range for inclusion
KSA-08-042	Date of genetically confirmed diagnosis of DMD is out of range for inclusion
KSA-09-009	Date of genetically confirmed diagnosis of DMD is out of range for inclusion

Table 14.3: Demographics and Medical History

Variable		n	%
**Age**	<2	2	0.9%
	≥2-<5	57	25.2%
	≥5-14	167	73.9%
	**n/nmiss**	**226/0**	
	Mean±sd	6.9±2.7	
	Median(Q1-Q3)	7.0(4.8, 8.5)	
	Min-Max	1.3-13.8	
	**n/nmiss**	**226/0**	
**Height**	Mean±sd	117.8±15.3	
	Median(Q1-Q3)	119.0(106.0, 126.5)	
	Min-Max	78.0-166.0	
	**n/nmiss**	**200/26**	
**Region Origin**	Makkah	45	20.0%
	Riyadh	42	18.7%
	Asser	20	8.9%
	Eastern Region	20	8.9%
	Madinah	17	7.6%
	Jazan	16	7.1%
	Northern Borders	14	6.2%
	Al Baha	13	5.8%
	Najran	12	5.3%
	Tabuk	10	4.4%
	Ha'il	7	3.1%
	Qassim	7	3.1%
	Al Jawf	2	0.9%
	**Total/nmiss**	**225/1**	
**Current Region**	Makkah	58	26.6%
	Riyadh	48	22.0%
	Eastern Region	30	13.8%
	Asser	17	7.8%

Variable		n	%
	Madinah	12	5.5%
	Northern Borders	12	5.5%
	Najran	10	4.6%
	Jazan	8	3.7%
	Tabuk	7	3.2%
	Qassim	6	2.8%
	Ha'il	5	2.3%
	Al Baha	4	1.8%
	Al Jawf	1	0.5%
	**Total/nmiss**	**218/8**	
**Ever been diagnosed with any medical condition other than DMD?**	Yes	23	10.2%
	No	203	89.8%
	**Total/nmiss**	**226/0**	
**Number of medical conditions**	0	203	89.8%
	1	18	8.0%
	2	5	2.2%
	**Total/nmiss**	**226/0**	
**Condition***	Acute Pancreatitis	1	0.4%
	Bronchial asthma	4	1.8%
	Attention Deficit hyperactivity disorder (ADHD)	2	0.9%
	Autism	2	0.9%
	Epilepsy	2	0.9%
	Obstructive sleep apnea	1	0.4%
	Beckwith Wiedemann syndrome	1	0.4%
	Bladder stones	1	0.4%
	Febrile seizure	1	0.4%
	GERD	1	0.4%
	Glucose 6 phosphate dehydrogenase deficiency	1	0.4%
	Hypertension	1	0.4%
	Hypotonia	1	0.4%
	Mild Aortic Regurgitation	1	0.4%
	Osteoporosis	1	0.4%
	Pericardial cyst	1	0.4%
	Pigeon-shaped chest protruded sternum	1	0.4%
	Right vesicoureteral reflux	1	0.4%
	Sickle cell anemia	1	0.4%
	Single kidney	1	0.4%
	Tonsillar hypertrophy	1	0.4%
	Tonsillectomy	1	0.4%

**Variable**		**n**	**%**
**Patients with Ongoing conditions**	Yes	22	9.7%
	No	204	90.3%
	**Total/nmiss**	**226/0**	
*Total number of conditions is 28 among 23 patients			

Table 14.4: Genetic Mutation Distribution

Variable	Retrospective (n=181)			Prospective (n=45)			Overall (n=226)		
	**n**	**%**	**95% Confidence interval**	**n**	**%**	**95% Confidence interval**	**n**	**%**	**95% Confidence interval**
Large deletion	104	57.5%	49.9%-64.8%	30	66.7%	51.0%-80.0%	134	59.3%	52.6%-65.8%
Large duplication	23	12.7%	8.2%-18.5%	2	4.4%	0.5%-15.1%	25	11.1%	7.3%-15.9%
Small Mutations*	54	29.8%	23.3%-37.1%	13	28.9%	16.4%-44.3%	67	29.6%	23.8%-36.1%
Nonsense mutations	35	19.3%	13.9%-25.9%	4	8.9%	2.5%-21.2%	39	17.3%	12.6%-22.8%
Deletion/insertion mutations	16	8.8%	5.1%-14.0%	9	20.0%	9.6%-34.6%	25	11.1%	7.3%-15.9%
Splicing mutations	3	1.7%	0.3%-4.8%	0	0.0%	0.0%-7.9%	3	1.3%	0.3%-3.8%
Other mutations	3	1.7%	0.3%-4.8%	0	0.0%	0.0%-7.9%	3	1.3%	0.3%-3.8%
**Total/nmiss**	**181/0**			**45/0**			**226/0**		
Patients with two or more small mutations	3	1.7%	0.3%-4.8%	0	0.0%	0.0%-7.9%	3	1.3%	0.3%-3.8%
*3 patients each had 2 small mutations									

Table 14.5: Patient Characteristics and Management Plan

Variable		Retrospective Patients (n=181)		Prospective Patients (n=45)		Total (n=226)	
		**n**	**%**	**n**	**%**	**n**	**%**
**Age at diagnosis**	Mean±sd	7.0±2.8		6.7±2.5		6.9±2.7	
	Median(Q1-Q3)	6.9(4.9, 8.6)		7.2(4.5, 8.2)		7.0(4.8, 8.5)	
	Min-Max	1.3-13.8		2.3-12.9		1.3-13.8	
	**n/ nmiss**	**181/0**		**45/0**		**226/0**	
**Age at first sign and symptoms**	Mean±sd	3.8±2.3		3.2±2.3		3.6±2.3	
	Median(Q1-Q3)	2.9(2.0, 4.9)		2.5(1.9, 3.5)		2.7(2.0, 4.6)	

**Variable**		**Retrospective Patients (n=181)**		**Prospective Patients (n=45)**		**Total (n=226)**	
	Min-Max	0.4-10.3		0.6-10.6		0.4-10.6	
	**n/nmiss**	**97/84**		**45/0**		**142/84**	
**Age at diagnosis**	<2	2	1.1%	0	0.0%	2	0.9
	≥2 - <5	44	24.3%	13	28.9%	57	25.2%
	≥5-14	135	74.6%	32	71.1%	167	73.9%
	**n/ nmiss**	**181/0**		**45/0**		**226/0**	
**Age at first sign and symptoms**	<2	21	21.6%	14	31.1%	35	24.6%
	≥2 - <5	54	55.7%	25	55.6%	79	55.6%
	≥5-14	22	22.7%	6	13.3%	28	19.7%
	**n/nmiss**	**97/84**		**45/0**		**142/84**	
**Site of diagnosis**	Current treatment center	143	79.0%	32	71.1%	175	77.4%
	Referring treatment center	31	17.1%	13	28.9%	44	19.5%
	Not Available	7	3.9%	0	0.0%	7	3.1%
**Year of Diagnosis (confirmed genetic test)**	2010	4	2.2%	0	0.0%	4	1.8%
	2011	3	1.7%	0	0.0%	3	1.3%
	2012	3	1.7%	0	0.0%	3	1.3%
	2013	12	6.6%	0	0.0%	12	5.3%
	2014	18	9.9%	0	0.0%	18	8.0%
	2015	22	12.2%	0	0.0%	22	9.7%
	2016	16	8.8%	0	0.0%	16	7.1%
	2017	24	13.3%	0	0.0%	24	10.6%
	2018	39	21.5%	0	0.0%	39	17.3%
	2019	26	14.4%	0	0.0%	26	11.5%
	2020	14	7.7%	7	15.6%	21	9.3%
	2021	0	0.0%	31	68.9%	31	13.7%
	2022	0	0.0%	7	15.6%	7	3.1%
	**n/nmiss**	**181/0**		**45/0**		**226/0**	
	2002	1	1.0%	0	0.0%	1	0.7%

Variable		Retrospective Patients (n=181)		Prospective Patients (n=45)		Total (n=226)	
**Year of first signs/symptoms**	2004	1	1.0%	0	0.0%	1	0.7%
	2007	2	2.1%	0	0.0%	2	1.4%
	2008	2	2.1%	0	0.0%	2	1.4%
	2009	8	8.2%	0	0.0%	8	5.6%
	2010	8	8.2%	0	0.0%	8	5.6%
	2011	6	6.2%	0	0.0%	6	4.2%
	2012	8	8.2%	0	0.0%	8	5.6%
	2013	8	8.2%	1	2.2%	9	6.3%
	2014	15	15.5%	3	6.7%	18	12.7%
	2015	8	8.2%	7	15.6%	15	10.6%
	2016	9	9.3%	8	17.8%	17	12.0%
	2017	10	10.3%	2	4.4%	12	8.5%
	2018	7	7.2%	6	13.3%	13	9.2%
	2019	4	4.1%	6	13.3%	10	7.0%
	2020	0	0.0%	6	13.3%	6	4.2%
	2021	0	0.0%	6	13.3%	6	4.2%
	**n/nmiss**	**97/84**		**45/0**		**142/84**	
**Referral from another center**	Yes	111	61.3%	28	62.2%	139	61.5%
	No	47	26.0%	17	37.8%	64	28.3%
	Not Available	23	12.7%	0	0.0%	23	10.2%
**Present walking characteristics**	Yes	150	82.9%	45	100.0%	195	86.3%
	No	5	2.8%	0	0.0%	5	2.2%
	Not Available	26	14.4%	0	0.0%	26	11.5%
**Type of present walking characteristics***	Not walking by 15 months.	56	37.3%	19	42.2%	75	38.5%
	Not walking by 18 months	46	30.7%	11	24.4%	57	29.2%
	Difficulty on walking	131	87.3%	40	88.9%	171	87.7%
	Walks on their toes	28	18.7%	16	35.6%	44	22.6%
	Waddles	54	36.0%	26	57.8%	80	41.0%

**Variable**		**Retrospective Patients (n=181)**		**Prospective Patients (n=45)**		**Total (n=226)**	
	Others	9	6.0%	2	4.4%	11	5.6%
**Patient Fully Wheelchair-Bound**	Yes	60	33.1%	4	8.9%	64	28.3%
	No	86	47.5%	41	91.1%	127	56.2%
	Not available	35	19.3%	0	0.0%	35	15.5%
	Missing	0	0.0%	0	0.0%	0	0.0%
**Age at Fully Wheelchair-Bound**	Mean±sd	10.5±2.4		9.7±1.6		10.4±2.3	
	Median(Q1-Q3)	10.7(9.3, 12.1)		9.0(8.6, 11.6)		10.6(9.1, 11.6)	
	Min-Max	5.0-15.5		8.6-11.6		5.0-15.5	
	**n/ nmiss**	**27/33**		**3/1**		**30/34**	
**Patient able to run. †**	Yes	38	44.2%	17	41.5%	55	43.3%
	No	30	34.9%	24	58.5%	54	42.5%
	Not available	18	20.9%	0	0.0%	18	14.2%
	Missing	0	0.0%	0	0.0%	0	0.0%
**Patient able to climb stairs. †**	Yes	51	59.3%	24	58.5%	75	59.1%
	No	26	30.2%	17	41.5%	43	33.9%
	Not available	9	10.5%	0	0.0%	9	7.1%
	Missing	0	0.0%	0	0.0%	0	0.0%
**Intellectual impairment**	Yes	46	25.4%	11	24.4%	57	25.2%
	No	97	53.6%	33	73.3%	130	57.5%
	Not available	38	21.0%	1	2.2%	39	17.3%
	Missing	0	0.0%	0	0.0%	0	0.0%
**Type of Intellectual impairment ‡**	**Low IQ**	**7**	**15.2%**	**0**	**0.0%**	**7**	**12.3%**
	**Speech delay**	**32**	**69.6%**	**5**	**45.5%**	**37**	**64.9%**
	**Learning difficulties**	**24**	**52.2%**	**6**	**54.5%**	**30**	**52.6%**
	**Autism-like behavior**	**1**	**2.2%**	**2**	**18.2%**	**3**	**5.3%**
	**Others**	**9**	**19.6%**	**2**	**18.2%**	**11**	**19.3%**
**Clinical Characteristics****	DMD associated cardiomyopathy	9	5.5%	0	0.0%	9	4.4%
	Enlargement of the Calves	137	84.0%	39	97.5%	176	86.7%

**Variable**		**Retrospective Patients (n=181)**		**Prospective Patients (n=45)**		**Total (n=226)**	
	Lumbar lordosis	65	39.9%	18	45.0%	83	40.9%
	Lumbar scoliosis	51	31.3%	15	37.5%	66	32.5%
	Trendelenburg gait	40	24.5%	19	47.5%	59	29.1%
	Sleep apnea	1	0.6%	0	0.0%	1	0.5%
	Other	7	4.3%	2	5.0%	9	4.4%
	N/Missing	**163/18**		**40/5**		**203/23**	
**Pulmonary Function Tests**	Yes	19	10.5%	2	4.4%	21	9.3%
	No	97	53.6%	43	95.6%	140	61.9%
	Not Available	65	35.9%	0	0.0%	65	28.8%
**FEV1 (in L)**	Mean/±sd	1.0±0.0		1.5±0.7		1.0±0.2	
	Median /(Q1-Q3)	1.0(1.0, 1.0)		1.5(1.0, 2.0)		1.0(1.0, 1.0)	
	Min/max	1.0-1.0		1.0-2.0		1.0-2.0	
**FVC (in L)**	Mean/±sd	1.1±0.2		1.5±0.7		1.1±0.3	
	Median /(Q1-Q3)	1.0(1.0, 1.0)		1.5(1.0, 2.0)		1.0(1.0, 1.0)	
	Min/max	1.0-2.0		1.0-2.0		1.0-2.0	
**FEV1/FVC (%)**	Mean/±sd	84.1±16.7		98.5±20.5		85.5±17.1	
	Median /(Q1-Q3)	78(75, 87)		98.5(84.0, 113.0)		79.0(75.0, 87.0)	
	Min/max	68.0-116.0		84.0-113.0		68.0-116.0	
**Predicted FVC (in L)**	Mean/±sd	4.5±14.7		36.0±49.5		7.5±20.1	
	Median /(Q1-Q3)	1.0(1.0, 1.0)		36(1.0, 71.0)		1.0(1.0, 1.0)	
	Min/max	1.0-65.0		1.0-71.0		1.0-71.0	
**ALT (in U/L)**	Mean/±sd	348.6±186.6		334.1±165.4		346.4±182.9	
	Median /(Q1-Q3)	332.0(223.0, 450.0)		327.0(221.0, 420.0)		331.0(222.0, 447.5)	
	Min/max	36.0-1181.0		81.0-746.0		36.0-1181.0	
	N/Missing	**115/66**		**21/24**		**136/90**	
**AST (in U/L)**	Mean/±sd	246.7±154.3		244.1±158.5		246.3±154.3	
	Median /(Q1-Q3)	211.0(142.0, 298.0)		189.5(127.0, 344.0)		210.0(134.0, 298.0)	
	Min/max	48.0-967.0		45.0-623.0		45.0-967.0	
	N/Missing	**99/82**		**18/27**		**117/109**	

Variable		Retrospective Patients (n=181)		Prospective Patients (n=45)		Total (n=226)	
**CK (in U/L)**	Mean/±sd	12585.3±7625.4		12336.6±7795.4		12546.7±7629.8	
	Median /(Q1-Q3)	11017.0(7560.0, 16955.0)		11350.0(5864.0, 15358.0)		11051.0(7560.0, 15844.0)	
	Min/max	759.0-50953.0		1100.0-29535.0		759.0-50953.0	
	N/Missing	**147/34**		**27/18**		**174/52**	
**Initial Management Plan**	Medication	129	72.9%	38	86.4%	167	75.6%
	Physical therapy	123	69.5%	34	77.3%	157	71.0%
	Physical Therapy plus Ataluren	6	3.4%	0	0.0%	6	2.7%
	Physical Therapy plus Steroids	59	33.3%	18	40.9%	77	34.8%
	Devices	64	36.2%	5	11.4%	69	31.2%
	Diet	23	13.0%	0	0.0%	23	10.4%
	Educational and Psychological Interventions	30	16.9%	7	15.9%	37	16.7%
	Surgery	1	0.6%	1	2.3%	2	0.9%
	Other	95	53.7%	38	86.4%	133	60.2%
	N/Missing	**177/4**		**44/1**		**221/5**	
**Medications**							
**Treatment**	Steroids Only	83	64.3%	21	55.3%	104	62.3%
	Ataluren Only	2	1.6%	0	0.0%	2	1.2%
	Steroids + Ataluren	5	3.9%	0	0.0%	5	3.0%
	Steroids + Eteplirsen	2	1.6%	0	0.0%	2	1.2%
	No Steroid/No Ataluren	37	28.7%	17	44.7%	54	32.3%
	n/no treatment/Missing	**129/48/4**		**38/6/1**		**167/54/5**	
**Supplements†***	Multivitamin	1	0.8%	0	0.0%	1	0.6%
	Calcium supplementations	41	31.8%	8	21.1%	49	29.3%
	Vitamin D	100	77.5%	37	97.4%	137	82.0%
	n/Missing	**129/48/4**		**38/6/1**		**167/54/5**	
**Other Medications†***	Esomeprazole magnesium	1	0.8%	0	0.0%	1	0.6%
	Omeprazole	8	6.2%	1	2.6%	9	5.4%

**Variable**		**Retrospective Patients (n=181)**		**Prospective Patients (n=45)**		**Total (n=226)**	
	Risperidone	1	0.8%	0	0.0%	1	0.6%
	Lisinopril dihydrate	2	1.6%	0	0.0%	2	1.2%
	n/Missing	**129/48/4**		**38/6/1**		**167/54/5**	
**Age at steroid initiation**	Mean/±sd	7.5±2.4		6.7±1.9		7.4±2.3	
	Median /(Q1-Q3)	7.3(5.9-8.7)		6.6(4.8-8.2)		7.1(5.7-8.7)	
	Min/Max	2.6-14.2		4.2-10.2		2.6-14.2	
	**n/nmiss**	**78/12**		**11/10**		**89/22**	
**Age group at steroid initiation**	≥2 to <5	13	16.7%	3	27.3%	16	18.0%
	≥5 to <7	22	28.2%	4	36.4%	26	29.2%
	>=7	43	55.1%	4	36.4%	47	52.8%
**Age at Ataluren initiation**	Mean/±sd	7.2±2.4				7.2±2.4	
	Median /(Q1-Q3)	7.5(5.7-8.8)				7.5(5.7-8.8)	
	Min/Max	2.9-9.9				2.9-9.9	
	**n/nmiss**	**6/1**				**6/1**	
**Age group at Ataluren initiation**	<7	2	33.3%			2	33.3%
	>=7	4	66.7%			4	66.7%
**Devices*** **	AFO	52	81.3%	1	20.0%	53	76.8%
	Bilateral AFO	1	1.6%	2	40.0%	3	4.3%
	Wheelchair	18	28.1%	0	0.0%	18	26.1%
	Medical Shoes	8	12.5%	2	40.0%	10	14.5%
	Medical insoles	2	3.1%	0	0.0%	2	2.9%
	Orthosis	2	3.1%	0	0.0%	2	2.9%
	Ankle Orthosis	1	1.6%	1	20.0%	2	2.9%
	Standing frame	1	1.6%	0	0.0%	1	1.4%
	Spinal brace	1	1.6%	0	0.0%	1	1.4%
	Bilateral knee immobilizer	1	1.6%	0	0.0%	1	1.4%
	Helmet	1	1.6%	0	0.0%	1	1.4%

**Variable**		**Retrospective Patients (n=181)**		**Prospective Patients (n=45)**		**Total (n=226)**	
	**n/no devices/missing**	**64/113/4**		**5/39/1**		**69/152/5**	
**Other Management plan†***	Cardiac Evaluation/Follow up/Management	91	51.4%	37	84.1%	128	57.9%
	Bone health care	20	11.3%	0	0.0%	20	9.0%
	Referral to pulmonary	5	2.8%	2	4.5%	7	3.2%
	Genetic referral/Carrier testing	3	1.7%	0	0.0%	3	1.4%
	Referral to occupational therapy	1	0.6%	0	0.0%	1	0.5%
	Referral to neurology	1	0.6%	0	0.0%	1	0.5%
	Speech Therapy	1	0.6%	0	0.0%	1	0.5%
	Nephrology consultation	1	0.6%	0	0.0%	1	0.5%
	n/Missing	**177/4**		**44/1**		**221/5**	

*Percentages computed among those who have present walking characteristics.
†percentages are computed among those who are not fully wheelchair bound.
‡Percentages are computed among those who had intellectual impairment.
**percentages don't add up to 100, as a patient might have multiple present clinical characteristics.
***The total number of patients with devices (69, 64 retrospective and 5 prospective) is given in the same table before, patients might have more than one device.
†*percentages are among those with management plans. Totals do not add up to the number as patients might have multiple management plans.
Imputations strategy: in case of incomplete dates, missing day will be replaced by 15th of the months, in case of missing months it will be imputed as June, and in case of missing day, month and year the date will be reported as missing.

Table 14.5.1: Steroid Therapy Among the Included Patients

	Steroid therapy (n=111)	
	n	%
Deflazacort	2	1.8%
Prednisolone	102	91.9%
Prednisone	7	6.3%

Table 14.6.1: Ambulation Characteristics of the Included Patients According to Types of Active Medication Received

		Type of Treatment					
		Steroid Only (n=104)		Steroid + Ataluren (n=5)		No Steroid or Ataluren (n=54)	
		n	%	n	%	n	%
Is the patient fully wheelchair-bound?	Yes	29	27.9%	1	20.0%	20	37.0%
	No	71	68.3%	4	80.0%	29	53.7%
	Not Available	4	3.8%	0	0.0%	5	9.3%
Is the patient able to run?*	Yes	30	42.3%	2	50.0%	13	44.8%
	No	30	42.3%	2	50.0%	11	37.9%
	Not Available	11	15.5%	0	0.0%	5	17.2%
Is the patient able to climb stairs?*	Yes	39	54.9%	4	100.0%	18	62.1%
	No	27	38.0%	0	0.0%	8	27.6%
	Not Available	5	7.0%	0	0.0%	3	10.3%

*Among those who are Not fully in Wheelchair

Table 14.6.1.1: Age at Fully Wheelchair Bound According to Types of Active Medication Received

Among those who are Wheelchair bound		Type of Treatment		
		Steroid Only (n=29)	Steroid + Ataluren (n=1)	No Steroid or Ataluren (n=20)
Age at fully wheelchair bound	Mean/±sd	10.3±2.3	10.8±0	9.6±2.7
	Median /(Q1-Q3)	10.1(9.3, 11.2)	10.8(10.8, 10.8)	9.8(8.0, 11.4)
	Min/Max	4.9-15.5	10.8-10.8	5.0-13.6
	n/nmiss	15/14	1/0	8/12

Table 14.6.2: Ambulation Characteristics of the Included Patients According to Age at the Start of Treatment

		Groups					
		Age at treatment <7 years (n=43)		Age at treatment ≥7 years (n=47)		Total (n=90)	
		n	%	n	%	n	%
Is the patient fully wheelchair-bound?	Yes	4	9.3%	23	48.9%	27	30.0%
	No	36	83.7%	23	48.9%	59	65.6%
	Not Available	3	7.0%	1	2.1%	4	4.4%
Is the patient able to run?*	Yes	17	47.2%	11	47.8%	28	47.5%
	No	10	27.8%	10	43.5%	20	33.9%
	Not Available	9	25.0%	2	8.7%	11	18.6%
Is the patient able to climb stairs?*	Yes	24	66.7%	16	69.6%	40	67.8%
	No	8	22.2%	6	26.1%	14	23.7%
	Not Available	4	11.1%	1	4.3%	5	8.5%

*Among those who are Not fully in Wheelchair

Table 14.6.3: Ambulation Characteristics of the Included Patients According to Age at Diagnosis

		Groups					
		Age at diagnosis <5 years (n=59)		Age at diagnosis ≥5 years (n=167)		Total (n=226)	
		n	%	n	%	n	%
Is the patient fully wheelchair-bound?	Yes	6	10.2%	58	34.7%	64	28.3%
	No	45	76.3%	82	49.1%	127	56.2%
	Not Available	8	13.6%	27	16.2%	35	15.5%
Is the patient able to run?*	Yes	23	51.1%	32	39.0%	55	43.3%
	No	12	26.7%	42	51.2%	54	42.5%
	Not Available	10	22.2%	8	9.8%	18	14.2%
Is the patient able to climb stairs?*	Yes	27	60.0%	48	58.5%	75	59.1%
	No	13	28.9%	30	36.6%	43	33.9%
	Not Available	5	11.1%	4	4.9%	9	7.1%

*Among those who are Not fully in Wheelchair

Table 14.7: Summary of Physical Therapy Management by Site and Steroid Treatment

Site*	Physical therapy= Yes n (%)				Physical Therapy= No n (%)			
	with Steroid (n=77)		without Steroid (n=80)		With Steroid (n=34)		Without Steroid (n=30)	
	n	%	n	%	n	%	n	%
KSA-01	18	23.4%	12	15.0%	7	20.6%	13	43.3%
KSA-02	4	5.2%	46	57.5%	0	0.0%	0	0.0%
KSA-03	10	13.0%	3	3.8%	7	20.6%	2	6.7%
KSA-04	3	3.9%	3	3.8%	3	8.8%	2	6.7%
KSA-05	0	0.0%	0	0.0%	0	0.0%	1	3.3%
KSA-06	18	23.4%	5	6.3%	1	2.9%	3	10.0%
KSA-07	18	23.4%	1	1.3%	2	5.9%	2	6.7%
KSA-08	4	5.2%	5	6.3%	12	35.3%	3	10.0%
KSA-09	2	2.6%	5	6.3%	2	5.9%	4	13.3%

*N=221 as 5 patients had no reported management plans

15. REFERENCE LIST

Al Jumah, M, Al Muhaizea, M, Al Rumayyan, A, Al Saman, A, Al Shehri, A, Cupler, E, et al. Current Managment of Duchenne Muscular Dystrophy in the Middle East: Expert Report. Neurodegener Dis Manag 2019;9(3):123-133.

Al Jumah, M, Majumdar, R and Al-Rajeh, S. Deletion Mutations in the Dystrophin Gene of Saudi Patients With Duchenne and Becker Muscular Dystrophy. Saudi Med J 2002;23(12):1478-1482.

AlSaman, AS, Al Ghamdi, F, Bamaga, AK, AlShaikh, N, Al Muqbil, M, Muthaffar, O, et al. Patient demographics and characteristics from an ambispective, observational study of patients with duchenne muscular dystrophy in Saudi Arabia. Front Pediatr 2022;10:1020059.

Birnkrant, DJ, Bushby, K, Bann, CM, Apkon, SD, Blackwell, A, Brumbaugh, D, et al. Diagnosis and Management of Duchenne Muscular Dystrophy, Part 1: Diagnosis, and Neuromuscular, Rehabilitation, Endocrine, and Gastrointestinal and Nutritional Management. Lancet Neurol 2018;17(3):251-267.

Bladen, CL, Salgado, D, Monges, S, Foncuberta, ME, Kekou, K, Kosma, K, et al. The TREAT-NMD DMD Global Database: Analysis of More Than 7,000 Duchenne Muscular Dystrophy Mutations. Hum Mutat 2015;36(4):395-402.

Bushby, K, Finkel, R, Birnkrant, DJ, Case, LE, Clemens, PR, Cripe, L, et al. Diagnosis and Management of Duchenne Muscular Dystrophy, Part 1: Diagnosis, and Pharmacological and Psychosocial Management. Lancet Neurol 2010a;9(1):77-93.

Bushby, K, Finkel, R., Birnkrant, D.J., Case, L.E., Clemens, P.R., Cripe, L., Kaul, A., Kinnett, K., McDonald, C., Pandya, S., Poysky, J.. . Diagnosis and management of Duchenne muscular dystrophy, Part 1: diagnosis and pharmacological and psychological management. The Lancet Neurology 2010b;9(1):77-93.

Chaudhary, AG, Alqahtani, M.H., Abuzenadah, A., et al. . Mutation Analysis in Saudi Duchenne and Becker Muscular Dystrophy Patients Using Multiplex PCRS. Arch Med Sci 2008;4(1):16-21.

Ellis, JA, Vroom, E and Muntoni, F. 195th ENMC International Workshop: Newborn Screening for Duchenne Muscular Dystrophy 14-16th December, 2012, Naarden, The Netherlands. Neuromuscul Disord 2013;23(8):682-689.

Emery, AE. Population Frequencies of Inherited Neuromuscular Diseases--A World Survey. Neuromuscul Disord 1991;1(1):19-29.

Friis, RH, Sellers, T.A. (2014). Eidemiology for Public Health Practice; Jones & Bartlett.

Gardner-Medwin, D, Bundey, S., Green, S. . Early Diagnosis of Duchenne Muscular Dystrophy. Lancet 1978;311(8073):1102.

Guo, R, Zhu, G., Zhu, H., Ma, R., Peng, Y., Liang, D., Wu, L. DMD Mutation Spectrum Analysis in 613 Chinese Patients With Dystrophinopathy. J Hum Genet 2015;60(8):435.

Koenig, M, Hoffman, EP, Bertelson, CJ, Monaco, AP, Feener, C and Kunkel, LM. Complete Cloning of the Duchenne Muscular Dystrophy (DMD) cDNA and Preliminary Genomic Organization of the DMD Gene in Normal and Affected Individuals. Cell 1987;50(3):509-517.

McDonald, CM, Henricson, EK, Abresch, RT, Duong, T, Joyce, NC, Hu, F, et al. Long-Term Effects of Glucocorticoids on Function, Quality of Life, and Survival in Patients With Duchenne Muscular Dystrophy: A Prospective Cohort Study. Lancet 2018;391(10119):451-461.

Mohammed, F, Elshafey, A, Al-Balool, H, Alaboud, H, Al Ben Ali, M, Baqer, A, et al. Mutation Spectrum Analysis of Duchenne/Becker Muscular Dystrophy in 68 Families in Kuwait. The Era of Personalized Medicine. PLoS One 2018;13(5).

Moxley, RT, 3rd, Pandya, S, Ciafaloni, E, Fox, DJ and Campbell, K. Change in Natural History of Duchenne Muscular Dystrophy With Long-Term Corticosteroid Treatment: Implications for Management. J Child Neurol 2010;25(9):1116-1129.

Mukoyama, M, Kondo, K, Hizawa, K and Nishitani, H, DMDR Group. Life Spans of Duchenne Muscular Dystrophy Patients in the Hospital Care Program in Japan. Journal of the Neurological Sciences 1987;81(2-3):155-158.

Nosaeid, MH, Mahdian, R, Jamali, S, Maryami, F, Babashah, S, Maryami, F, et al. Validation and Comparison of Two Quantitative Real-Time PCR Assays for Direct Detection of DMD/BMD Carriers. Clin Biochem 2009;42(12):1291-1299.

Patterson, V, Morrison, O., Hicks, E. . Mode of Death in Duchenne Muscular Dystrophy. Lancet 1991;337(8744):801-802.

Prior, TW and Bridgeman, SJ. Experience and Strategy for the Molecular Testing of Duchenne Muscular Dystrophy. The Journal of Molecular Diagnostics 2005;7(3):317-326.

Sbiti, A, El Kerch, F and Sefiani, A. Analysis of Dystrophin Gene Deletions by Multiplex PCR in Moroccan Patients. Biomed Res Int 2002;2(3):158-160.

Tayeb, MT. Deletion Mutations in Duchenne Muscular Dystrophy (DMD) in Western Saudi Children. Saudi J Biological Sciences 2010;17(3):237-240.

Todorova, A, Todorov, T, Georgieva, B, Lukova, M, Guergueltcheva, V, Kremensky, I, et al. MLPA Analysis/Complete Sequencing of the DMD Gene in a Group of Bulgarian Duchene/Becker Muscular Dystrophy Patients. Neuromuscul Disord 2008;18(8):667-670.

Venugopal, V, Pavlakis, S., Schaefer, L., Meager, M. Duchenne Muscular Dystrophy. StatPearls 2019.

Vry, J, Gramsch, K, Rodger, S, Thompson, R, Steffensen, BF, Rahbek, J, et al. European Cross-Sectional Survey of Current Care Practices for Duchenne Muscular Dystrophy Reveals Regional and Age-Dependent Differences. J Neuromuscul Dis 2016;3(4):517-527.

Wang, Y, Yang, Y, Liu, J, Chen, XC, Liu, X, Wang, CZ, et al. Whole Dystrophin Gene Analysis by Next-Generation Sequencing: A Comprehensive Genetic Diagnosis of Duchenne and Becker Muscular Dystrophy. Mol Genetics Genomics 2014;289(5):1013-1021.

Yiu, EM, Kornberg, A.J. Duchenne Muscular Dystrophy. Journal of Paediatrics and Child Health 2015;51(8):759-764.

Zhang, T, Liu, S., Wei, T., Yong, J., Mao, Y., Lu, X., Xie, J., Ke, Q., Jin, F., Qi, M. Development of a Comprehensive Real-Time PCR Assay for Dystrophin Gene Analysis and Prenatal Diagnosis of Chinese Families. Clinica Chimica Acta 2013;424:33-38.

Signature Page for PTC-GD-MA-405 Study Report v1.0

Medical Affairs Approval	Christian Werner<br>Medical Affairs Approval<br>I approve the document(s)<br>31-Oct-2023 16:27:48 GMT+0000

Signature Page for VV-CLIN-012473 v1.0
